# A Systematic Screen for Micro-RNAs Regulating the Canonical Wnt Pathway

**DOI:** 10.1371/journal.pone.0026257

**Published:** 2011-10-17

**Authors:** Roman Anton, Sujash S. Chatterjee, Julia Simundza, Pamela Cowin, Ramanuj DasGupta

**Affiliations:** 1 Cancer Institute/Department of Pharmacology, New York University Langone Medical Center, New York University, New York, New York, United States of America; 2 Departments of Cell Biology and Dermatology, New York University Langone Medical Center, New York University, New York, New York, United States of America; Childrens Hospital Los Angeles, United States of America

## Abstract

MicroRNAs (miRs) and the canonical Wnt pathway are known to be dysregulated in human cancers and play key roles during cancer initiation and progression. To identify miRs that can modulate the activity of the Wnt pathway we performed a cell-based overexpression screen of 470 miRs in human HEK293 cells. We identified 38 candidate miRs that either activate or repress the canonical Wnt pathway. A literature survey of all verified candidate miRs revealed that the Wnt-repressing miRs tend to be anti-oncomiRs and down-regulated in cancers while Wnt-activating miRs tend to be oncomiRs and upregulated during tumorigenesis. Epistasis-based functional validation of three candidate miRs, miR-1, miR-25 and miR-613, confirmed their inhibitory role in repressing the Wnt pathway and suggest that while miR-25 may function at the level of â-catenin (β-cat), miR-1 and miR-613 act upstream of β-cat. Both miR-25 and miR-1 inhibit cell proliferation and viability during selection of human colon cancer cell lines that exhibit dysregulated Wnt signaling. Finally, transduction of miR-1 expressing lentiviruses into primary mammary organoids derived from Conductin-lacZ mice significantly reduced the expression of the Wnt-sensitive β-gal reporter. In summary, these findings suggest the potential use of Wnt-modulating miRs as diagnostic and therapeutic tools in Wnt-dependent diseases, such as cancer.

## Introduction

Colon and gastrointestinal cancers are amongst the leading causes of cancer-related mortality and they all have been linked, together with many other cancers, to mutations in components of the Wnt/β-catenin pathway [1]. Therefore there is a major interest in targeting the activity of this pathway using genetic and chemical therapeutic tools. The promise of one emerging approach rests upon the therapeutic potential of small interfering RNAs (siRNAs) and microRNAs (miRs). miRs are small RNAs (∼ca. 22 nt in length) that regulate the level of mRNAs and proteins by targeted degradation of specific mRNAs and/or repression of their translation [2],[3]. Functions of miRs have been identified in apoptosis, proliferation, differentiation [2] and stem cell maintenance [4]. They have also been associated with cancer progression and metastasis [5],[6],[7]. Steady-state expression profiles of certain miRs have been often found to be deregulated in cancers and can aid in prognosis [8],[9],[10]. Individual miRs that have been reported to down-regulate oncogenes such as ras [11] are called anti-oncomiRs and inhibit cancer proliferation. Others, termed oncomiRs, function in a cancer-supportive or inductive manner by down-regulating tumor-suppressors such as p53 [12],[13] and inducing proliferation and/or metastasis. The canonical Wnt/β-catenin pathway is often found to be elevated in gastrointestinal, breast and colon cancers among others and there is strong evidence for a role of hyper-activated Wnt signaling in cancer initiation and progression [14],[15],[16],[17],[18]. The key element of Wnt signaling is the transcriptional co-activator role of β-catenin, whose level is tightly controlled by a destruction complex including a scaffold protein, Axin-1, APC, and GSK-3β, a kinase that phosphorylates β-catenin, which results in its ubiquitination and subsequent proteasomal degradation [18],[19]. Wnt signaling via LRP5/6/Frizzled receptors and cytosolic Dsh among other factors, destabilizes this destruction complex, which leads to accumulation of β-catenin and its association with TCF/LEF family transcription factors in the nucleus to activate specific target genes [18],[19]. Negative regulators of Wnt signaling like APC and Axin function as tumor-suppressors and the viability of some cancer cell lines is believed to be Wnt-dependent [20],[21],[22],[23],[24],[25].

It has been recently suggested that the delivery and use of anti-oncomiRs or inhibiting oncomiR functionality with antagomiRs [26] may serve as a promising therapeutic approach [27]. We therefore hypothesized that identifying and characterizing miRs that specifically modulate the canonical Wnt pathway could provide a basis for the development of novel Wnt-based therapeutics in Wnt-associated diseases, such as cancer. Research in the past few years have implicated some miRs in the regulation of Wnt signaling [28],[29],[30],[31],[32],[33],[34]. Here we report a systematic screening of a library of 470 human synthetic Pre-miRs and identification of 38 miRs that modulate the activity of the Wnt pathway in human HEK293 cells. Secondary validation and functional testing of 3 candidate miRs, namely miR-1, miR-25 and miR-613 confirmed their inhibitory effect on the activity of the Wnt pathway. Epistasis experiments revealed that miR-1 and miR-613 target the pathway upstream of Axin or active β-catenin, and that miR-25 acts downstream, at the level of β-cat, likely by targeting β-cat's coding sequence. Importantly, overexpression of miR-25 and miR-1 inhibited proliferation/viability of human colon cancer cells that are known to be dependent on sustained β-cat signaling for their survival [22],[24]. Furthermore, expression of miR-1 in primary mammary epithelial organoids derived from a Wnt-reporter mouse (conductin-lacZ) significantly reduced the expression of the β-gal reporter. These results suggest that these candidate miRs may influence Wnt signaling activity *in vivo*.

## Methods

### Screening and reporter assays

Screening and reporter assays were carried out as described previously [35]. Briefly, HEK293 cells trypsinized and resuspended in antibiotic-free culture media were plated and transfected in 384 well plates (Corning, Cat No. 3704). Transfections were conducted with 0.1 µL Lipofectamine 2000 (Invitrogen) and 27 ng STF19 reporter plasmid (a kind Gift from the R.T. Moon laboratory, Seattle, USA) together with 5 ng Renilla-CMV vector as internal control each well. 7 µL DNA-containing transfection-mix with serum-free DMEM and Lipofectamine2000 was added to the pre-plated Hsa-pre-miR™ in a 384 well plate (5 µL each well, 1.5 pmol, human pre-miR library, n = 470, Ambion, #4385830) for a final concentration of 42.9 nM ca. 5000 viable HEK293 cells were plated in 23 µL culture media to the pre-dispensed transfection mixes and incubated for two days. 50 µL of pretested Wnt3a-conditioned media (harvested from L-Wnt3A cells [36], a kind gift from the laboratory or Dr. R. T. Moon, University of Washington, Seattle) were added at day 2 post-transfection and cells were incubated for additional 16 hours. Pre-miRs™ used are synthetic siRNA-like and modified strand selective small dsRNAs with verified specificity (Ambion). Cells were lysed in 20 µL DualGlo (Promega, #E2920) substrate buffer and normalized luminosity, which is the ratio of the firefly reporter (STF16x-Firefly) and the renilla luciferase (CMV-driven), was read with an EnVision multilabel plate-reader (Perkin Elmer). The ratio of the STF-firefly and CMV-renilla RLU (relative light units) for each Pre-miR was divided by the plate average (PA) or control siRNAs, respectively. The primary screen was performed in quadruplets for statistical/assay robustness. Cutoff values were based on the performance of control siRNAs with the only exception of the high/medium-scorer miR-200a that was also included into the cherrypick-listing because of its described role [29],[32]. Raw data, normalized values, and cherry-picking listings are deposited available at the NYU-RNAi core facility, available upon request, and found in the supplementary information (Fig. S9). Other reporter assays, including epistasis experiments, were conducted in 96- or 384-well plates using the same protocol settings. STF19/CMV-RL activity in cancer cell lines was measured one day post-transfection.

### Cloning, DNA and RNA reagents

The coding region of human β-catenin (S37A mutant; transcript nt position 307–1874 NcoI-Klenow; NotI) was subcloned into the 3′UTR of the Renilla gene (NotI, SpeI-Klenow) within a psi-check-2 reporter vector with modified MCS to monitor the influence of miR-25 on its transcript stability and translation. In this assay a CMV-driven synthetic-firefly-luciferase served as internal control. A β-catenin fragment lacking exon-3 (543–1874) behaved similar (not shown). A human Pri-miR-25 fragment was PCR amplified from human genomic DNA (HEK293 cells) with the following primer pair: FP-miR-25: 5′-gcggccgccattctcagacgtgcctaag-3′, RP-miR-25: 5′-tctagatgattacc-caacctactgct-3′. After sub-cloning the hsa-Pri-miR-25 amplicon that contains endogenous 5′- and 3′-flanking sequences was finally cloned into the pcDNA3.1(-) vector (Invitrogen) via BamHI (vector and insert) and NheI (vector) XbaI (insert). HPLC grade human synthetic Pre-miR™ precursor miRNAs that are strand-selection optimized/approved and chemically modified siRNA-like precursor miRs (Pre-miR-1™ #AM17100; Pre-miR-25™ #AM17100; Pre-miR-613™ #AM17100) were purchased from Ambion. Three Axin1 and Axin2 Stealth siRNA (Invitrogen, Cat. No.: 119026-B03, -B05, -B01, -C07, -C11, -C09) were used as positive control and epistatic-inductors to activate the canonical Wnt pathway at its downstream components. Three Stealth siRNAs for β-catenin (Invitrogen, 119026B07/B09/B11) were used to down-regulated Wnt activity as positive control for a Wnt-pathway inhibitory RNA. Silencer-Negative Control siRNA #4 (Ambion, Invitrogen #AS00K2L1) and Silencer-Negative control siRNA #2 (Ambion, Invitrogen #AS00JSIG) were used as negative controls. The Pre-miR™ precursor molecule library was plated in 5 µL with 1.5 pmol each well into two 384 well plates at the RNAi screening core facility using an automated dispenser system (Janus MDT, Perkin Elmer) and stored at −80°C. pLV-miR-1 and pLV-miR-control lentivirus were obtained from Biosettia (San Diego, USA).

#### Bioinformatics, and statistical analysis

Sequences were aligned with ClustalW, visualized and processed with Genedoc software, and Treepuzzle and Treeview software were employed for similarity and phylogenetic analysis (parameters: 10.000 puzzling steps; quartet puzzling tree reconstruction, neighbor-joining tree, approximate quartet likelihood, HKY-model of substitution). miRNA target prediction and alignment blasts were done with miRWalk, Pictar, Diana-microT, PITA, RNAhybrid, Target Scan, miRanda, NCBI-blasts. Statistical analysis: Z-factors were calculated with Z = 1-(3(σ_p_+σ_n_)/(|μ_p_-μ_n_|)) and values between 0.5 and 1.0 indicated good screening parameters. Log-Z-score was deployed on log-tansformed (Nexp FF/RL) data set. Z = ((FF/RL)_LOG_-PA_LOG_)/STDEV(P_LOG_); FF, TCF19x-firefly RLU; RL, CMV-Renilla RLU; PA, plate average; RLU, relative light units; STDEV(P) standard deviation of the plate. Local plate averages (7–10 data points) were used for alternative balancing of normalized data of the primary screen. Regulation of transcript level/translation is measured by the psi-check-2 reporter and determined by relative changes of RLU values via R(r) = (RL-β-cat_CDS_/FF)/(RL-empty/FF); with R(r) of controls = 1. Pubmed (NCBI) and Google searches were used for data mining to find cancer relevant reports on miRs identified and verified for correlation studies.

#### Western blotting

SDS-PAGE and western blotting was performed with standard protocols including TBST (0.1%) washing buffer and 4% BSA TBST blocking buffer. Primary antibodies (anti-β-catenin, Sigma, #C7207, anti-tubulin (α-Tubulin), Sigma, #T9026) were incubated over night while gently shaking at 4°C in 1∶1000 to 1∶2000 dilutions. Infrared (IR)-dye conjugated secondary antibodies (1∶20.000, goat anti-mouse, IRDye™800, #610-132-121, Rockland) were incubated at room temperature for 1 h in blocking buffer and subsequently rinsed with TBST. Blots were visualized with the Odyssey infrared imaging system and quantified with the Odyssey software (Li-Cor, Biosciences). For semi-quantitative western blotting the average of the ratios of β-catenin and α-Tubulin (α-TUB) [signal intensity] was divided by the average of the ratios of the control experiments.

#### Cell culture and cell lines

HEK293 human embryonic kidney cells (ATCC, cat # CRL-1573), HCT116 human colon cancer cells (ATCC, cat # CCL-247), SW480 human colon cancer cells (ATCC, cat # CCL-228), and MCF7 human breast cancer cells (ATCC, cat # HTB-22) were cultured in filtered DMEM media supplemented with 10% fetal calf serum, 1 mM L-glutamine and 1× non-essential amino acids (ATCC, #203166) without antibiotics at 37°C and 5% CO_2_. Selection media for cells transfected with linearized empty pcDNA3.1(-) or Pri-hsa-miR-25 pcDNA3.1(-) using Lipofectamine2000 contained increasing amounts of active G418 sulfate (Cellgro #30-234-CR) for 7–16 days. Lenti-pLV-miR-1 and -pLV-control transduced cells were selected with puromycin for up to 7 days.

#### RT-qPCR

Real-Time relative quantification PCR was conducted with 25 µL iTAQ™ SYBR-Green Supermix (1×) with ROX (BIO-RAD, #20361) and the qPCR thermocycler Mx3005P (Stratagene) system including the MxPro-Mx30055 v.4.10 Build 389 software (Stratagene) and the delta-delta-C_t_ calculation method. Thermocycler conditions: 96°C initial denaturation step for 10 min, 50 cycles of (96°C denaturation for 30 s, 57°C annealing for 30 s and 72°C elongation for 10 s). Amplification and dissociation curves revealed the specificity of the qPCR products, which were also examined by DNA agarose TAE gel electrophoresis (2% agarose, with EtBr). Unmodified exon-spanning primer pairs with similar annealing temperature (57°C) and product size (80–110 bp) were used. Total RNA was isolated with RNeasy (Qiagen) and cDNA was synthesized using the High-capacity cDNA reverse transcription kit (Applied Biosystems, #4368814). Briefly, 1 µg of total RNA was DNaseI digested, heat inactivated, and input for cDNA reverse transcription in 20 µL following the instructions of the manufacturer. Primer sequences: CTNNB1-fw 5′-ATGGCAACCAAGAAAGCAAG-3′; CTNNB1-rv 5′-GGTCCACAGTAGTTTTTCGTAAG-3 (product size 101 bp); internal control primer: GAPDH-fw 5′-TGAAGGTCGGAGTCAACG-3′, GAPDH-rv 5′-GGGTCATTGATGGCAACA-3′ (product size 97 bp).

#### *In vivo* context analysis of the regulation of axin2/Conductin-lacZ reporter by miR-1

Two *axin2/Conductin*-lacZ mature female mice [37] were sacrificed and mammary epithelial organoids prepared essentially as described [38]. Six mammary glands/mouse were collected in DMEM/F12 10% FBS medium on ice. The glands were transferred to a sterile Petri dish and minced into a homogenous paste using a pair of sterile scalpels. The minced tissue was transferred to a 15 ml falcon tube containing 9 ml Epicult–B Basal Medium (Stem Cell Technologies) and 1 ml of 10× collagenase and hyaluronidase (3000 and 1000 units/ml Stem Cell Technology) and constantly agitated (environmental shaker) for 1 h at 37°C. The tissue was then collected by centrifuged at 450 g for 10 min and re-suspended in cold HBSS 2% FBS. The organoids were isolated from contaminating fibroblasts and blood cells by 2 s pulse centrifugation at 450 g (repeated 5–7 times) their purity was assessed by examination on a glass slide. The final pellet was re-suspended in 2 ml Trypsin/EDTA (0.25%) and incubated at 37°C for 1 min. The sample was then diluted in 10 ml of cold HBSS containing 2% FBS and spun down at 450 g for 5 min. The supernatant containing stringy DNA was removed carefully and the remaining pellet was re-suspended in pre-warmed fresh dispase containing 500 µl (2000 unit/ml) DNase, mixed for 1 min and spun down. Organoids were then re-suspended and plated in Epicult-B Basal Medium. Control and miR-1 expression vectors (pLV-miR-1 from Biosettia Inc., USA) were used for lentiviral transduction of the cells in 24-well plates. After 12 h of incubation, the cells were fixed in 4% formaldehyde (Electron Microscopy Sciences) for 20 min, washed 3 times with 1× PBS, permeabilized and blocked with blocking buffer (0.1% Triton X-100, 1% BSA, 5% normal goat serum in DPBS) at room temperature for 20 min and immuno-stained with mouse anti-β-GAL 1∶50 (DHSB, 4°C overnight), anti-mouse IgG-Alexa488 goat antibody (Invitrogen; 1∶1000; 2 h at room temperature) and DAPI. The cells were imaged using a Nikon TE2000PFS and the levels of β-gal (fluorescence intensity) was measured by the NIS elements software (Nikon, USA).

## Results

### Identification of human microRNAs in a primary Wnt-reporter screen

**Figure 1:**
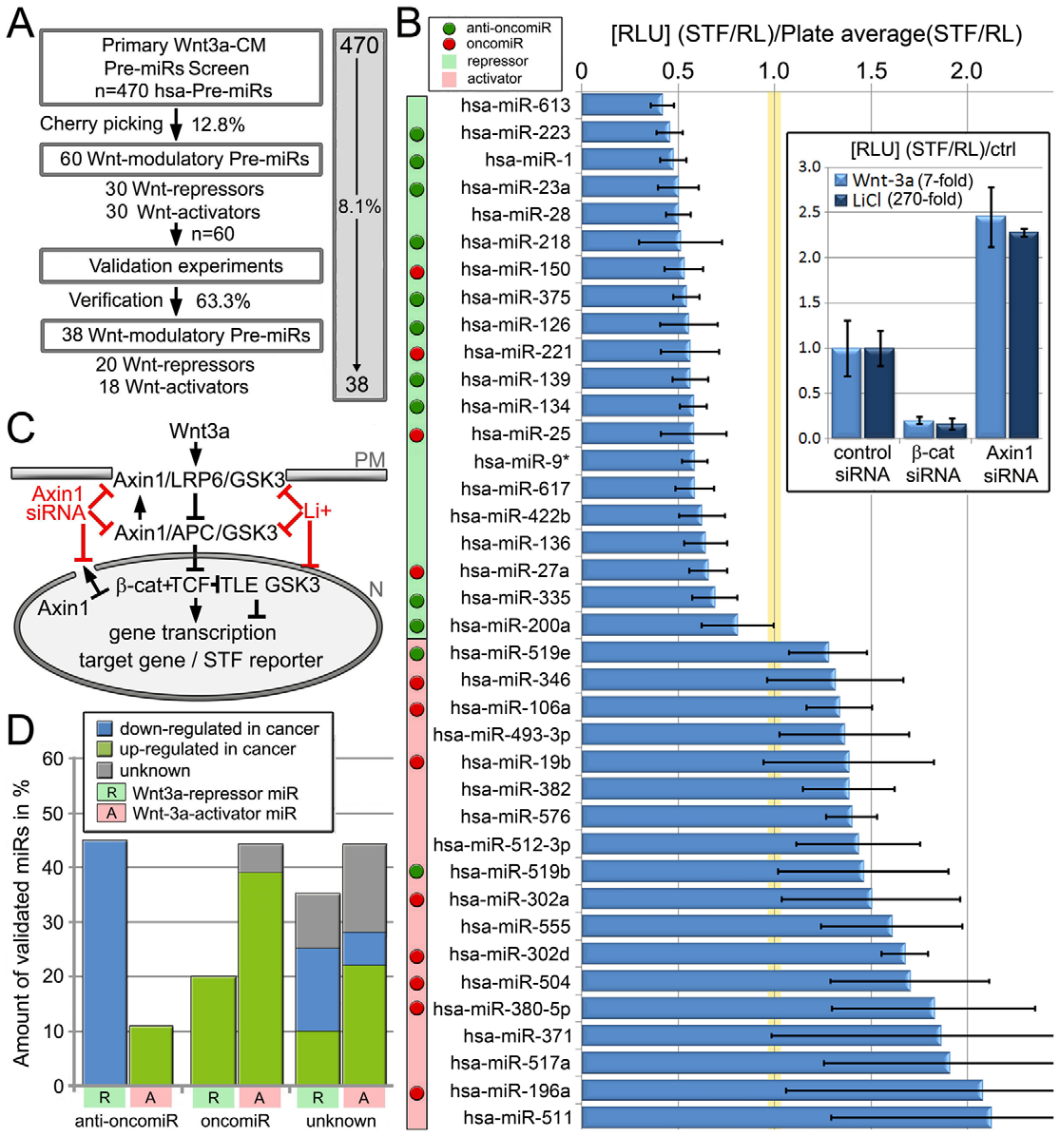
Validation of the Pre-miR/Wnt3a/STF-report screen in HEK293 cells. (A) Schematic overview of the screening and validation procedure. (B) Target listing of all validated synthetic cherry-pick Hsa-Pre-miRs and the average of all measurements with SEM (N = 2, n = 8). Color-code: Hsa-Pre-miRs that significantly repressed (green tab) or activated (red tab) the STF reporter of canonical Wnt-pathway activity. Green disc: anti-oncomiR; red disc: oncomiR. Inset: Representative performance of control siRNAs in one of the assays with LiCl or Wnt3a pathway induction. (C) Simplified sketch of important Wnt/β-catenin pathway nodes/switches and its activation sites by axin1/2 siRNAs or LiCl (D) Correlation between Wnt-pathway repressor (R) and activator (A) miRs being anti-oncogenic (anti-oncomiRs) or oncogenic (oncomiRs). Within each column the fraction of Pre-miRs whose expression is up- or down-regulated in cancer is indicated by color.

Micro-RNAs (miRNAs, miRs) and the Wnt pathway play fundamental roles in development and disease by regulating expression of target genes. We hypothesized that certain miRs could fine-tune the activity of β-catenin-dependent “canonical” Wnt signaling by modulating the abundance of relevant pathway components. To address this question we performed a luciferase reporter screen in human HEK293 cells to investigate the regulatory capacity of a library of 470 miRs on the activity of the canonical Wnt pathway. HEK293 cells were transiently co-transfected with individual synthetic Pre-miR™s, a luciferase gene reporter driven by 16 functional TCF/LEF1 binding sites and a CMV-Renilla luciferase that served as internal control for cell viability and transfection efficiency. The canonical Wnt pathway was activated with Wnt3a-conditioned media from L-cells 2.5 days post-transfection, and plates were read after 16 h of Wnt3a-CM treatment at day 3. The primary screening was performed in quadruplets in a 384-well plate format and yielded a set of 60 (of 470) candidate Wnt-regulatory human miRNAs (30 activators/synergists and 30 repressors), representing 12.8% of the strand-selective, synthetic Pre-miR™ Library (Ambion) (Fig. 1A, Fig. S1). A sector of the averaged and normalized screening results and a representation of the Z-score of the entire log-transformed data set is shown in Fig. S1 (B, C). The pCMV-Renilla-normalized Wnt/miR-screening results are available in the supplementary material S9. Following verification of consistent “hit” reproducibility, in a second round of low-throughput screening, 38 of the original 60 Pre-miRs were validated as unidirectional regulators, representing a validation rate of 63.3% which is about 8% of the entire library (Fig. 1A, B). Positive controls with β-catenin siRNAs, Axin1/Axin2 siRNA, and negative siRNA controls showed expected and reproducible results (Fig. 1 B, inset). The z-factor for the high throughput screen (HTS) was determined to be z>0.55 suggesting a robust setup for HTS and was also utilized for cut-off designations together with the normalized values of all positive controls.

### Wnt-modulation by miRs correlates with miR-oncogenicity and sequence similarity

**Figure 2:**
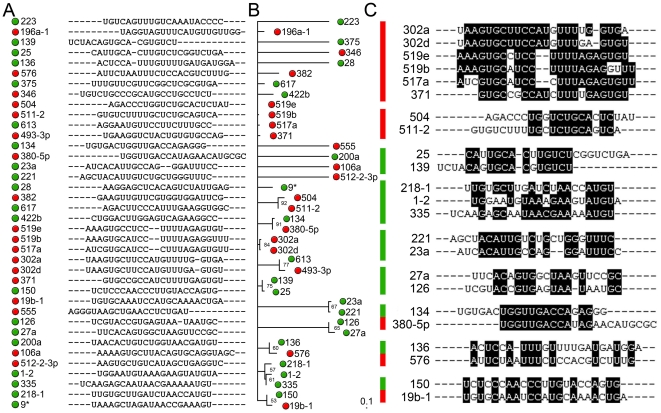
Alignment and tree of all validated Wnt-regulating Pre-miRs to represent and visualize sequence similarities. Numbers indicate Pre-miR designation. I. e. 233 stands for the mature hsa-Pre-miR-223 strand. Green: Pre-miR that was identified and verified as Wnt-inhibitory in the STF reporter assay. Red: Pre-miR that was identified as Wnt-activator/synergizer (A) Alignment of all mature strand selected verified Hsa-Pre-miRs (B) Quartet puzzling tree phylogram determined with tree-puzzle to visualize the degree of sequence similarities between all Pre-miRs based on nucleotide substitutions (C) Representative examples of miRs that share a high degree of sequence similarities. Note the enrichment of sequence similarity with unidirectional Wnt pathway modulation (see also Fig. S3).

Having determined the initial list of verified Wnt-modulating miRs (Fig. 1B), we examined possible correlations between the candidate miRs. An alignment of all mature miRs revealed a significant enrichment of sequence similarities within all validated miRs, some of which are members of the same family. Notably, we found that sequence similarity between miRs correlates with their ability to either activate or inhibit the activity of the Wnt-pathway (Fig. 2). An unbiased alignment of all identified mature miR sequences, shown in Fig. 2A, indicates several obvious consensus sequence conservations amongst all validated candidate miRs. MiRs with high sequence similarity can be also easily identified in a quartette puzzling tree, Fig. 2B, where nucleotide substitution is a measure of branch length like in phylogenetic trees. Wnt-repressing miRs are indicated by a green disc and Wnt-activators are indicated by a red disc. Thus it became apparent that high sequence similarity often goes in parallel with unidirectional modulation of the Wnt pathway (Fig. 2B). Most obvious and interesting sequence similarity examples are also shown in Fig. 2C. Specifically, this correlation or trend was very clear for 6 miRs, miR-302a,-302d, -519e, -519b, -517a, and -371 e.g.; they all hyper-activated the Wnt-reporter and happen to exhibit high sequence similarity (Fig. 2C). Of these, miR-302a and miR-302d are members of the miR-302 family and miR-519e, -519b, and -517a belong to the miR-515 family. A closer look at sequence similarity analysis of the whole miR-515 family revealed a striking consensus sequence between related Wnt-activating miRs (Fig. S8). Moreover, this consensus sequence is also highly shared by the miR-activators of the miR-302 family. Hsa-miR-371 is part of the miR-290 family, in agreement with alignments and phylogenetic Stem Loop (SL)-miR trees (Fig. S3), but also inherits, what we would suggest, a newly identified “Wnt-activator associated signature” (GUGCNNCCN(N)(N)UUU(N)NNG). Curiously, this activator consensus sequence is discontinuous and consists of the 3′-half of the major and highly conserved miR-seed sequence and a more 5′-part of a “co-seed” sequence, which is part of the mature miR and 3′-downtstream of the major-seed. This consensus sequence could thus play a role in target mRNA recognition in addition to its putative function in the formation of the loop structure of the stem-loop miR precursor. A comparison of Pre-miR-SL and mature miR sequence alignments and trees also helps to discern conserved versus non-conserved/adopted consensus and seed sequences (Fig. S3). Importantly, the majority of these similar mature miRs and SL-miRs shows a modulation of the canonical Wnt pathway in the same direction (Fig. S3, inset), which could indicate a causal relationship between sequence similarity/specificity and similar function.

Interestingly, further data mining via literature searches revealed another outstanding and surprising coherency that also supported our initial hypothesis: a majority of the miRs that have been reported to be anti-oncogenic and are repressed in cancers displayed Wnt-inhibitory properties, whereas those described as oncogenic miRs and are often elevated in cancers were identified as activators of the Wnt pathway by trend (Fig. 1D, for references see Table in Supplementary Fig. S6).

### Secondary validation of miR-1, miR-25 and miR-613

We chose to further investigate three candidate miRs that repressed Wnt3a/β-catenin signaling in the primary screen: miR-1, miR-25 and miR-613. Phylogenetic analysis support the miR-base classification that miR-1 belongs to the miR-1/206 family including hsa-miR-206 and the *Drosophila* dme-miR-1, an indication of the high evolutionary conservation of this family (shown in Supplementary, Fig. S2A by alignments and phylogenetic quartette puzzling trees); hsa-miR-25 belongs to the evolutionary conserved miR-25/92 family [39] including *Drosophila* miR-92a+b/310/311/312/313, shown in Fig. S2B, and shares only the seed sequence with other miRs like miR-4325 or miR-367. MiR-613 showed the strongest inhibition in the primary validation assay (Fig. S1) and shares the same seed sequence as the miR-1/206 superfamily (5′-GGAAUGU-3′) but shows no additional overall conservation (Fig., S4). On the other hand miRs like miR-32 or 367 that shares the seed with the miR-25/92 family could not repress Wnt reporter activity (Fig.S4). This could indicate that some miRs or miR-families may repress the Wnt pathway components/activity mainly with the seed sequence (miR-1/613-family), while others may require the coordinated action of the seed and co-seed (miR-25/92-family).

**Figure 3:**
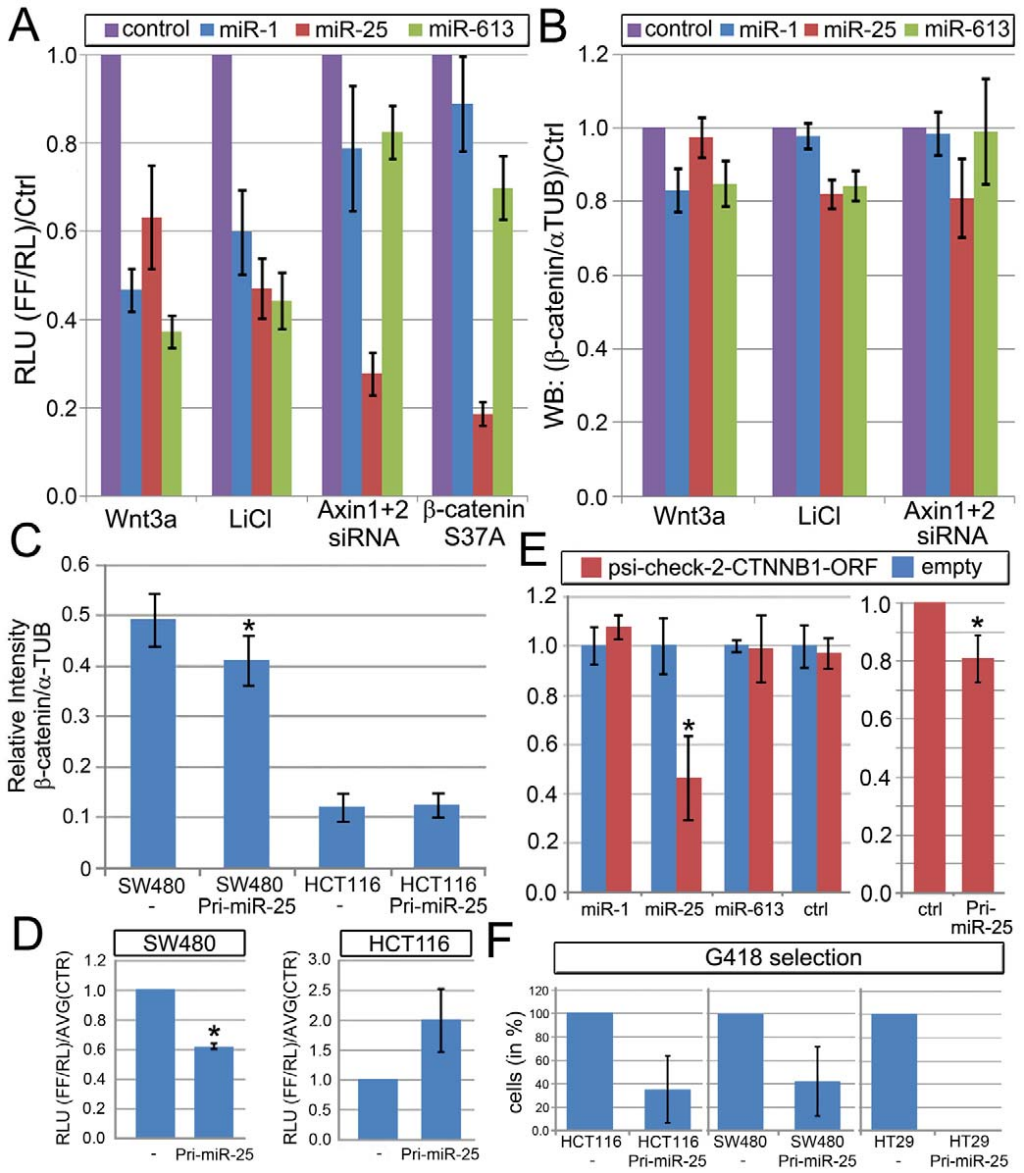
Molecular and functional characterization of candidate Wnt-regulatory miRs. (A) Epistasis experiments with synthetic human Pre-miR-1, Pre-miR-25 and Pre-miR-613. Influence of miRs on different epistatic pathway stimulations with Wnt3a, LiCl, Axin(1+2)-siRNA or S37A stabilized β-catenin that escapes default ubiquitination and degradation. (B) Semi-quantitative Western blotting to measure relative total β-catenin protein level changes (β-catenin normalized with α-Tubulin level) in HEK293 cells transfected with 50 nM Pre-miR-1, Pre-miR-25 or Pre-miR-613 at day 3 using different pathway inductions (for 1 day) as indicated. (C) Semi-quantitative Western blotting measurement of relative total β-catenin protein level changes in SW480 colon cancer cell (APC deficiency). (D) STF19x reporter assay normalized by CMV/Renilla to measure endogenous pathway activity in Pri-miR-25 stable cancer cell lines (SW480, HCT116) compared to empty vector control stable cells. (E) Psi-check2 reporter assay system to measure the influence of β-catenin CDS mRNA fragment (inserted into the 3′UTR of a Renilla gene) on transcript translation in the presence of indicated miRs. Left: Blue columns represent the empty vector control. Red columns indicate the psi-check2-CTNNB1-CDS vector. Right: normalized psi-check2-CTNNB2 vector values are shown. (F) Viability of transfected cancer cells during G418 selection. Relative amount of cells obtained after the selection procedure to establish HCT116, SW480, and HT29 colorectal cancer cell lines expressing Pri-miR-25-pcDNA3.1(-)-Neomycin compared to empty vector control cells. Asterisk: unpaired student's t-test, (P<0.05).

To understand at which step these miRs (miR-1,-25,-613) modulate the linear cascade of the Wnt pathway, and to identify their potential target genes, a series of epistasis experiments were conducted in HEK293 cells using the Wnt reporter and different pathway activators (Fig. 3 A, B). Induction of the Wnt pathway with Wnt3a-CM or by the GSK3β-inhibitor Lithium salt (LiCl) could be repressed by all three miRs. Elevated reporter activity by simultaneous siRNA mediated knockdown of Axin1 and Axin2 could be strongly inhibited by transfection of Pre-miR-25 (P<0.05; unpaired t-test), while miR-1 and miR-613 showed no significant influences (P>0.05). While Axin1 and GSK3β occupy similar complexes at the plasma membrane with LRP6, and with β-catenin in the cytosolic destruction complex, their functions may diverge elsewhere, such as in the nucleus where Axin-1 is also thought to be involved in nuclear export mediated repression of β-catenin function [40],[41]. These observations suggest an additional role of Axin downstream/independent of GSK3β. In agreement with this, Axin1/2 siRNAs could further synergize pathway activation in Wnt3a (7-fold activity) and LiCl (270-fold activity) induced cells to a very similar extent (Fig. 1 B inset), indicating that Axin inhibition, activates the pathway additively irrespective of the level of GSK3β-inhibition. Additionally, only miR-25 inhibited the activity of degradation-resistant S37A β-catenin mutant on the STF reporter (Fig. 3 A). Taken together, these data suggest that miR-25 represses the Wnt pathway downstream of GSK3β, Axin1/2 and stabilized β-catenin, while miR-1 and miR-613 act upstream of Axin1/2 and stabilized β-catenin but probably downstream of LiCl-mediated inhibition of GSK3β.

Relative quantification with real time quantitative PCR (RT-qPCR) did not reveal any significant reduction in β-catenin (CTNNB1) mRNA level in HEK293 cells (Fig. S7), nor for BCL9, Lef-1, TCF-1 (TCF7), Pygo1, Pygo2, Maml1, TCF-3, (TCF7l1) TCF-4 (TCF7l2) and western blotting also revealed no change for TCF-4 (not shown). While miR-1 and miR-613 could slightly reduce Wnt3a-CM mediated induction of β-catenin protein levels in HEK293 cells, miR-25 and miR-613 expression resulted in a moderate (∼20%) reduction in LiCl induced total β-catenin protein level, (Fig. 3B).

The epistasis experiments indicated that miR-25 may act in parallel or downstream of β-catenin itself. Intriguingly the most stringent RNAhybrid predictions that allow non-canonical seed sequences indicated some potential binding sites of miR-25 in the β-catenin cDNA (Fig. S5), while other target prediction algorithms (PicTar, EMBL-Microcosm, etc.) did not predict β-catenin as a target, because there is no distinct site in its 3′UTR. To test whether miR-25 could directly target the β-catenin cDNA, a fragment of the β-catenin coding sequence (CDS) containing the potential miR-25 binding sites was cloned into the psi-check-2 reporter. Renilla gene activity with an inserted β-catenin CDS in the 3′UTR indicated a significant miR-25-dependent reduction while control siRNAs, miR-1 or miR-613 had no effect (Fig. 3E). Notably, not only synthetic Pre-miR-25 but also over-expression of Pri-miR-25 could reduce a normalized Renilla gene activity with the β-catenin-CDS-fragment-3′UTR but to a lesser degree. As RT-qPCR experiments revealed no significant change in β-catenin transcript levels for all three miRs (Fig. S7), one remaining possibility is that miR-25 represses translation of β-catenin but not its transcript level. As β-catenin is predominantly and tightly regulated via post-translational regulation it is likely that translational reduction might play a minor role in uninduced or moderately induced cells, such as by Wnt3a-CM which induces reporter activity by only 7-10-fold (Fig. 3B); while during extensive pathway activation via LiCl, which can induce a >200-fold induction in the STF-assay, translational differences could play an increasingly relevant and important function in modulating the steady-state protein levels and are thus then detectable in our assays.

### Investigating functions of miR-1 and miR-25 in Wnt-relevant colon cancer cell lines

In order to investigate the function of miR-25 in Wnt-responsive cell lines we cloned a human unprocessed Pri-miR-25 into the pcDNA3.1(-) expression vector with a selectable neomycin marker. Upon transfection of colon cancer cells (HCT116, HT29, SW480) with Pri-miR-25 expressing vector, the number of Pri-miR-25 stable cell-colonies was markedly reduced compared to empty vector controls (Fig. 3F). This was particularly evident in HT-29 cells where despite several attempts we could not generate HT29-pcDNA3.1(-)-hsa-miR-25 expressing stable cell lines, although a few pcDNA3.1(-) empty vector transfected clones survived the selection procedure (Fig. 3F). HT29 cells are known to be dependent on APC-deficiency induced β-catenin activity for their survival, as reintroduction of a cDNA coding for a wild-type APC induces apoptosis [22], which could explain our observation. Additionally, expression of Pri-miR-25 in SW480 colon cancer cells, that exhibit high Wnt/β-catenin activity due to an APC truncation, significantly inhibited both the STF Wnt-reporter activity by ∼40% (Fig. 3D) and β-catenin protein levels by 20% (Fig. 3C). However HCT116 colon cancer cells that are known to exhibit high levels of endogenous Wnt pathway activity due to an intrinsic mutation in the β-catenin gene (ΔS45) showed no reduction in the STF and β-catenin expression levels.

**Figure 4:**
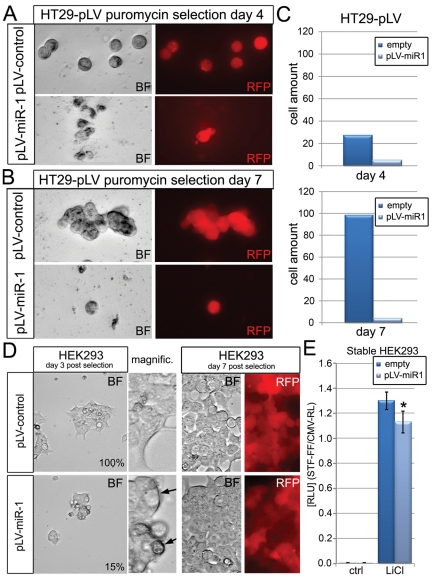
Characterization of miR-1 overexpression in HT29 and HEK293 cells. (A) HT29 colon cancer cells expressing pLV-Hsa-Pre-miR-1 or control vectors at day 4 of puromycin selection. (B) HT29 cells expressing pLV-Hsa-Pre-miR-1 or control at day 7 of puromycin selection. (C) Quantification of HT29 cells at day 4 and 7. RFP (red) fluorescence indicates the expression of RFP harboring the intronic miR-fragment. (D) HEK293 cells expressing miR-1 or control at day 3 (left) and day 7 (right) post puromycin selection. RED RFP fluorescence indicates expressing HEK293 cells. (E) LiCl induced Wnt pathway activity in stable miR-1expressing HEK293 cells.

We also investigated the potential function of the candidate Wnt-inhibitor miR-1 in the Wnt-dependent HT29 cancer cell line, because miR-1 was identified as one of the strongest repressors of Wnt-3a-induced activation of the STF reporter (Fig. 4). HT29 cells stably expressing intronic miR-1 in the 5′-UTR of rPURO, a red fluorescent puromycin-N-acetyl-transferase, were generated. These cells express red fluorescence and exhibit puromycin resistance. Hsa-miR-1 expressing cells displayed markedly reduced viability at day 4. While control-virus infected HT29 cells exhibited normal proliferation and colony-formation efficiency at day 7, Pre-miR-1 expressing HT29 cells did not show any obvious signs of proliferation (Fig. 4A–C). HEK293 cells that stably express hsa-miR-1, while showed an initial reduction in cell proliferation at day 4 of selection compared to control, did not display any major proliferation defect by day 7. These results may suggest that the compromised viability in HT29 cells, as compared to HEK293 cells, can be due to a specific Wnt-dependence of HT29 colon cancer cells for their survival (Fig. 4D).

### miR-1 inhibits expression of a Wnt-responsive reporter (conductin-lacZ) in primary mammary organoids

**Figure 5:**
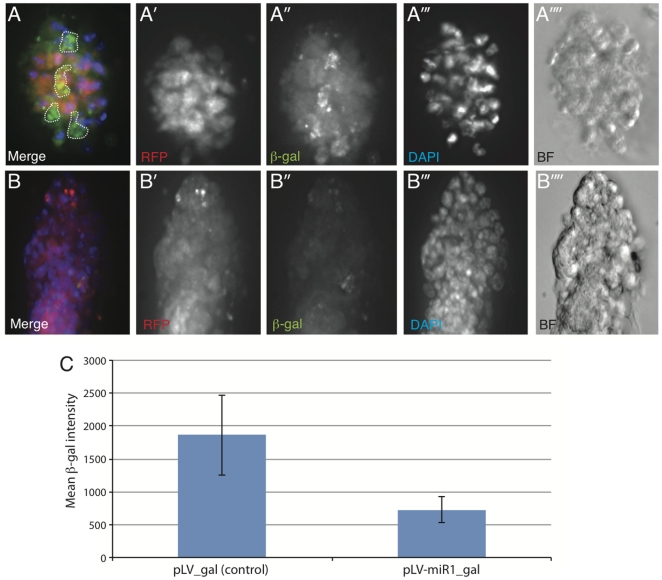
Lentiviral expression of miR-1 inhibits expression of the Wnt reporter, *axin2/Conductin*-LacZ in primary mouse mammary epithelial organoid cultures. (A-A″″) Representative image of organoid transduced with pLV-dsRed (Control) vector. (B-B″″) Representative image of organoid transduced with pLV-miR-1-dsRed lentiviral vector. The organoids transduced with control lentiviral vector (A″) shows significantly higher expression of Axin2-β-gal compared to organoids expressing miR-1 (B″). The dotted lines in panel A represent non-cellular auto-fluorescence, which was excluded from analysis of fluorescence intensity measured by NIS Elements Software. (C) Quantitative measurement of mean levels of β-gal (fluorescence intensity) in six individual organoids transduced with pLV-dsRed (Control) and seven individual organoids transduced with pLV-miR1-dsRed lentiviral vector. The average intensities were calculated for Region of interest (ROI) selected for each organoid using dsRed expression (that represents lentiviral infection) using the NIS Elements Software. Error bars denote standard deviation among samples.

To test whether candidate miRs can influence Wnt signaling in an *in vivo* context we derived primary mammary epithelial organoids from the *axin2*/conductin-lacZ mouse [37] using protocols described in Teissedre et al. [38]. Conductin-lacZ has been previously shown to respond to activated-Wnt signaling in mammary epithelial tissue. We introduced a miR-1 expression construct into mammary epithelial organoids derived from the conductin-lacZ *in vivo* reporter mouse using lentiviral transduction (pLV-miR-1 from Biosettia Inc., USA) and investigated whether expression of miR-1 could influence the expression of the β-gal reporter compared to pLV-empty vector control. As shown in Fig. 5, while pLV control vector (as followed by DsRED expression) did not influence the expression of β-gal reporter (green) (Fig. 5A-A″″), expression of miR-1 within the organoids strongly repressed reporter activity (Fig. 5B-B″″) (see quantification in Fig. 5C). In fact in most cases, miR-1 transduced organoid colonies exhibited almost no immuno-reactivity towards β-gal under identical exposure conditions. These data strongly suggest that ectopic expression of miR-1 may be sufficient in inhibiting the Wnt-responsive reporter in an *in vivo* context.

## Discussion

In this report we provide the results from a comprehensive screen for the identification, and validation/characterization of human Wnt pathway-modulating miRNAs using a systematic HTS approach. Three candidate Wnt-repressing miRs, namely, miR-1, miR-25 and miR-613, were further characterized in cell-biological assays. In addition to the known Wnt-modulatory miRNAs, such as miR-200a [29],[32], Drosophila miR-315 [28], miR-8 [30], miR-27 [31], zebrafish miR-203 [33], and miR-34 [34], we identified 37 additional miRs that modulate the activity of the Wnt-pathway reporter in cultured human cells. The functions of these 37 miRs, their potential evolutionary conservation, as well as their putative target genes can now be addressed in future studies.

Notably, we uncovered an interesting correlation between many of the candidate miRs that exhibit sequence similarities, both within and outside their mature seed sequence, and their ability to exert similar modulating influence on the activity of the Wnt3a/β-catenin pathway, thereby indicating sequence specificity in miR-mediated Wnt-pathway modulation. The consensus sequences may indicate novel functional seed and “co-seed” sequences that may be involved in the modulation of the Wnt pathway. The partly disrupted consensus seed and co-seed sequences might reflect the imperfect base-pairing with their cognate target genes that participate in Wnt signaling. Moreover, alignment of miRs that could regulate the Wnt reporter made intra- and inter-family related functional consensus sequences apparent (i.e. the seed of miR-1 and miR-613 or within the miR-302 and -515 families (Fig. S8)). This allows the generation of testable hypotheses regarding the functional relevance of specific nucleotide substitutions in miRs within the same family (Fig. S4).

Interestingly, our data also revealed that Wnt-inhibitory miRs tend to be anti-oncomiRs and Wnt-activating miRs tend to be oncomiRs. While the target-directness of each miR needs to be identified and validated in future studies, these data suggest that oncomiRs could contribute to elevated Wnt-pathway activity in cancers (Fig. 1), whereas anti-oncomiRs could function in buffering high levels of Wnt activity by keeping the expression of pathway components in check. Deregulated miR expression profiles might also contribute to oncogenesis by repressing the tumor suppressors, p21/p53 (miR-25 [42],[43], miR-504 [44]) which in turn might affect Wnt signaling. Disruption of cell cycle regulation may also stem from deregulated Wnt activity [45]. As the Wnt pathway is known to be pro-proliferative, and has target genes like c-myc and cyclin-D1 [24],[46], in certain contexts including cancers, the fine-tuning or buffering of Wnt activity by several distinct miRs could modulate proliferative potential of tumor cells, which might also explain the observed correlation. Further studies addressing the identities of the direct targets of candidate miRs can clarify their precise function(s) in the regulation of the Wnt pathway in oncogenesis. This could be addressed in global biochemical approaches such as SILAC or AGO-RIPA (also via HITS/PAR-CLIP [47],[48]) in cytosolic DGCR8 or Drosha loss of function cells transfected with synthetic miRs to identify direct targets.

3 out of 38 candidate miRs (miR-1, miR-25, miR-613) were further characterized in Wnt-responsive cultured cells and all were validated for their Wnt-inhibitory properties identified in the initial screen. Epistasis experiments revealed that candidate miRs target the signaling network at different sites. Pre-mir-1 may function most upstream, followed by miR-613 and then miR-25, which seems to influence the most downstream activity at the level of β-catenin. All miRs down-regulated Wnt3a-CM and LiCl induced Wnt pathway activity, while only Pre-miR-25 was able to repress Axin1+2-siRNAs or β-catenin-S37A induced activity. Hsa-Pre-mir-1 had a lesser ability to reduce total β-catenin protein levels under conditions of high pathway activation with LiCl. Interestingly, the results from this set of epistasis experiments are in agreement with a known function of Axin downstream/independent of the GSK3 β-catenin destruction complex (Fig. 1C), probably via nuclear-export mediated reduction (Fig. 1B, inset). Hsa-miR-1 and -613 seem to be not closely/directly related but share identical seed sequences and act upstream of Axin and probably downstream of GSK3 (as judged by their inhibitory effect on LiCl mediated activation of the reporter). Therefore both miRs may target a component of the Wnt-pathway upstream of β-catenin via their identical seed sequence, although the precise and relevant molecular targets remain to be identified. That said, it is important to note that cMET has been previously suggested to be a direct target of miR-1 [49],[50],[51].

Analyses of miR-25 function in the regulation of the Wnt pathway suggests a potential function in the translational inhibition of β-catenin via its binding to the β-catenin coding sequence and not its 3′-UTR. Expression of miR-25 repressed the psi-check2 sensor containing the miR-25 binding site, and moderately reduced β-catenin protein levels, while β-catenin transcript levels remained unchanged. Curiously the effect of hsa-miR-25 on β-catenin seems to be more effective under conditions of high pathway and low destruction complex activity, when translational differences preponderate and come into play due to strongly reduced post-translational regulation of β-cat. Reduced β-cat protein amounts can thus be better resolved in LiCl-induced HEK293 cells with high pathway activity, while a low pathway activity by Wnt-3a-CM (ca. 10-fold in STF assay, LiCl ca.50–100-fold) could only be reduced by miR-1 and miR-613 that can block a more upstream part of the pathway and are thus more efficient (Fig. 3D). A downstream role of miR-25 is also in agreement with a Drosophila miR-25/92 evolutionarily related cluster (Fig. S2) that can target the Wnt/Wg pathway (Pancratov and DasGupta, *unpublished data*). Recent evidence also suggests that miR-25 may inhibit Wnt/β-catenin dependent cancer viability by targeting Pcaf [52] which binds, acetylates, stabilizes and activates β-cat [53], thereby corroborating our observation that the influence of miR-25 is likely at the level of β-cat. Intriguingly, miR-32, that inherits the same seed as miR-25 also targets Pcaf [52] but instead, upregulates the Wnt-reporter in our reporter assays (Fig. S4). These observations suggest that the coding sequence of β-catenin itself may be the primary and direct target of miR-25. Additionally, the opposite effects of miR-25 and miR-32 on the Wnt reporter may imply an important and distinguishing role for the co-seed sequence of miR-25, which is strongly divergent between miR-25 and miR-32 (Fig. S4).

Finally, the very strong anti-proliferative effect of hsa-miR-1 in Wnt/β-catenin dependent human cancer cells (HT29) but not in HEK293 cells, combined with its strong inhibitory effect on an *in vivo* Wnt-reporter in primary mammary epithelial organoids (Fig. 5), and its lack of known oncogenic properties, highlight its potential as a novel miRNA-based candidate for the development of anti-cancer therapies. Upstream inhibitors like miR-1 besides downstream inhibitors like miR-25 thus show interesting properties for anti-cancer treatments in Wnt-dependent cancers and further support current findings that upstream components of the Wnt pathway are also valid and rational targets for cancer-therapies, even in cells with downstream mutations [15],[54],[55].

In summary our study reports the first comprehensive identification of Wnt-modulating miRs in human cells and represents how miR-based HTS can be employed as a powerful tool to systematically identify pathway relevant miRs. Since pathway-modulatory miRs could have functional impact in cancers associated with deregulated cell signaling, these findings could also benefit the long-term goal of developing miR-based therapeutics and for the diagnostic classification of cancers by expression profile signatures.

## Supporting Information

Figure S1 Results **of the primary Wnt/miR screen.** (A) Summarized target listing of cherrypick miRs identified as Wnt-modulating in a primary STF19x-based reporter screen in HEK293 cells. (B) Representative section of the primary screen showing the average of quadruplets as normalized values (STF19x TCF-sites firefly divided by CMV-driven Renilla internal control). Identified target miRs are indicated. (C) Graph showing sorted Z-score values of logarithmized screen data for better comparability. (D) Z-Score of log-transformed screen data (LOG-Z) (green), linear regression on values of unchanged values (red), its subtraction form LOG-Z, and division by 0.56 for a better visual representation (blue). Estimation of about 160 (34%) Wnt-modulating miRs with noise exceeding z-score average values, 80 activators and 80 repressors, respectively. Please note: Anticipating a validation rate of 63.3% (see main text) would yield 101 miRs in the library that modulate the Wnt pathway, representing 21,5%.
(TIF)

Figure S2 **Alignment and phylogenetic quartet puzzling trees of investigated miR families** (A) Mature and stem-loop miR strand alignment and tree of members of the miR-1/206 family. (B) Mature and stem-loop alignment of members of the miR-25/92 family.
(TIF)

Figure S3 **Alignment and phylogenetic quartet puzzling tree of all validated Wnt3a-modulating stem-loop miRs identified.** (A) Phylogenetic tree of the alignment. Light green: Inhibitor Stem-Loop (SL)-miR similarity group. Light-red: activating SL-miR similarity group. Inset: Quantification of SL- and mature miRs that show the same effect on the canonical Wnt pathway. (B) Alignment of all validated Wnt-modulating SL-miR sequences using ClustalW. (C) Comparison and visualization of the similarity groups identified for stem-loop and mature miRs and their effect on the canonical Wnt pathway.
(TIF)

Figure S4 **Screening results and alignment of studied miRs (miR-1/206 and miR-25/92 family) and miRs with a similar seed sequence.** (A) STF19x/CMV-RL reporter values measured in the primary screen for miR-1/206 related miRs inheriting the GGAAUGU seed sequence and their alignment. (B) STF19x/CMV-RL reporter values measured in the primary screen for miR-25/92 related miRs inheriting the UUGCAC seed sequence and their alignment. Note that a few nucleotide substitutions could affect modulation of the Wnt-pathway as measured within the primary screen.
(TIF)

Figure S5 **Possible miR-25 binding sites in β-catenin CDS (543–1874) predicted with RNAhybrid and without seed sequence constraints to include non-canonical seed identification.** Mfe: minimum free energy is indicated.
(TIF)

Figure/Table S6 **Table with all data mining references for the correlation studies of identified human Wnt-regulatory miRs and their oncogenicity.** (T1.R) Oncogenicity of validated miR/Wnt-repressors. (T1.A) Oncogenicity of validated miR/Wnt-synergizer miRs. (T2.R) Expressional changes of validated miR/Wnt-repressors in cancer. (T2.A) Expression changes of validated miR/Wnt-synergizers in cancer.
(DOCX)

Figure S7 **Q-PCR result for β-catenin mRNA levels in Hek293 cells transfected with 50 nM of indicated synthetic Pre-miRs or control siRNAs in the presence of 20 mM LiCl.** Note: No significant changes of β-catenin mRNA levels could be measured for all miRs tested.
(TIF)

Figure S8 **Extraction of a consensus sequence of identified miRs within the miR-515 and miR-302 family.** Alignment to identify a functional consensus in the miR-515 family and its overlap with modulators the miR-302 family in regarding their ability to modulate the Wnt pathway. (A) Alignment of all tested miR-515 family members in the primary Wnt/miR-screen. (B) Tree of related miR-sequences with normalized Wnt/miR-screen values and standard deviations indicated (local plate average corrected). (C) Alignment of all isolated Wnt-synergizing miR-515 family members to identify a functional RNA consensus sequence that could be essential for the activation-potential on the canonical Wnt pathway. Identification of a common consensus sequence between the regulatory miR-515 and miR-203 family members (below).
(TIF)

Figure S9 **Primary screen data including Nexp (STF-19x/CMV-renilla), p-values, standard deviation, averaged Nexp-values and list of estimated regulatory miRs.** A graph illustrates validated miRs within the whole screen data (Nexp values).
(XLSX)
