# Supplementary figures and images for: A Systematic Screen for Micro-RNAs Regulating the Canonical Wnt Pathway
Source: PLoS One. 2011 Oct 17;6(10):e26257. doi: 10.1371/journal.pone.0026257 (PMC3197157; doi:10.1371/journal.pone.0026257)

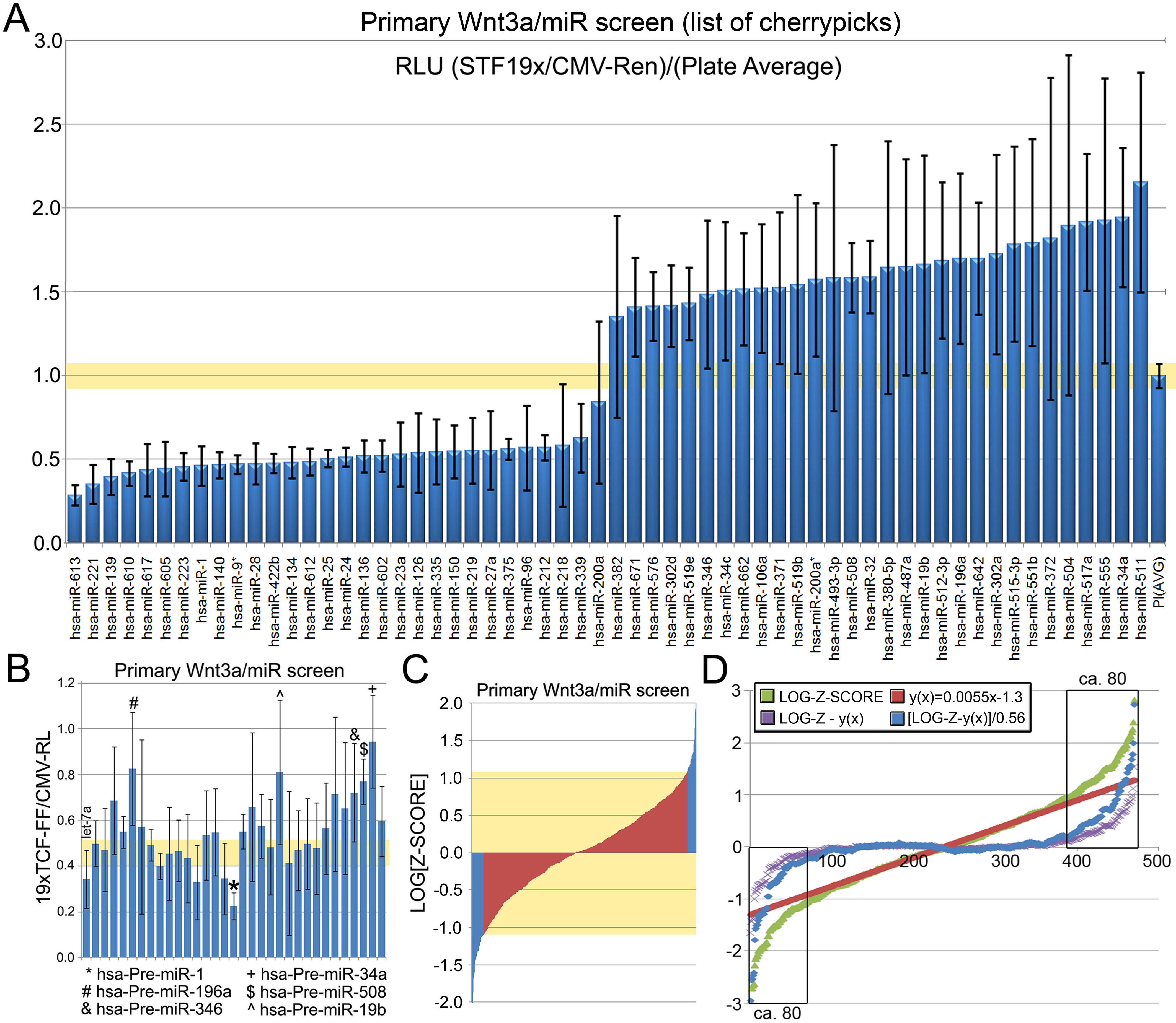

Supplement: Figure S1 — Results of the primary Wnt/miR screen. (A) Summarized target listing of cherrypick miRs identified as Wnt-modulating in a primary STF19x-based reporter screen in HEK293 cells. (B) Representative section of the primary screen showing the average of quadruplets as normalized values (STF19x TCF-sites firefly divided by CMV-driven Renilla internal control). Identified target miRs are indicated. (C) Graph showing sorted Z-score values of logarithmized screen data for better comparability. (D) Z-Score of log-transformed screen data (LOG-Z) (green), linear regression on values of unchanged values (red), its subtraction form LOG-Z, and division by 0.56 for a better visual representation (blue). Estimation of about 160 (34%) Wnt-modulating miRs with noise exceeding z-score average values, 80 activators and 80 repressors, respectively. Please note: Anticipating a validation rate of 63.3% (see main text) would yield 101 miRs in the library that modulate the Wnt pathway, representing 21,5%. (TIF) [file pone.0026257.s001.tif]

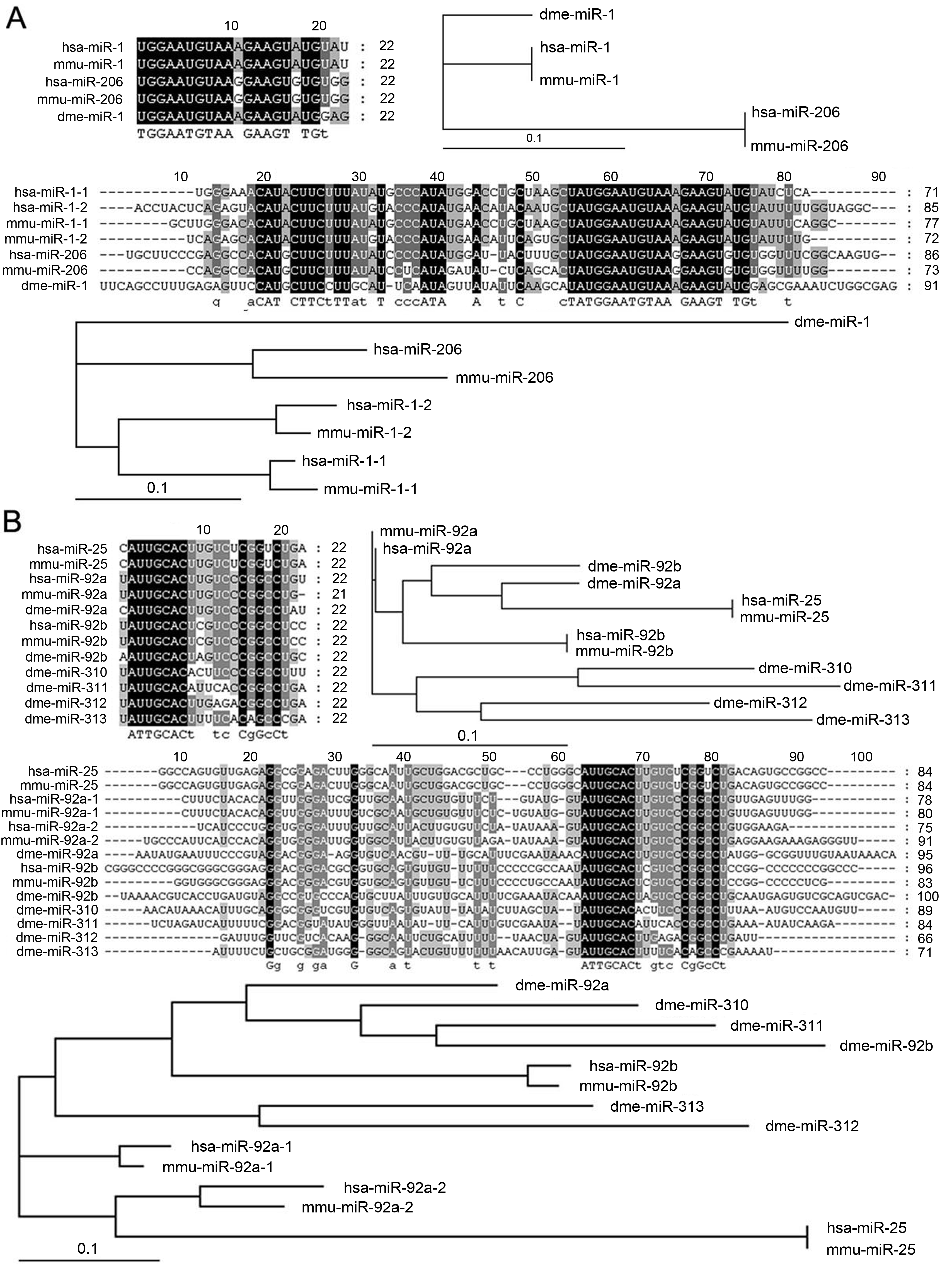

Supplement: Figure S2 — Alignment and phylogenetic quartet puzzling trees of investigated miR families (A) Mature and stem-loop miR strand alignment and tree of members of the miR-1/206 family. (B) Mature and stem-loop alignment of members of the miR-25/92 family. (TIF) [file pone.0026257.s002.tif]

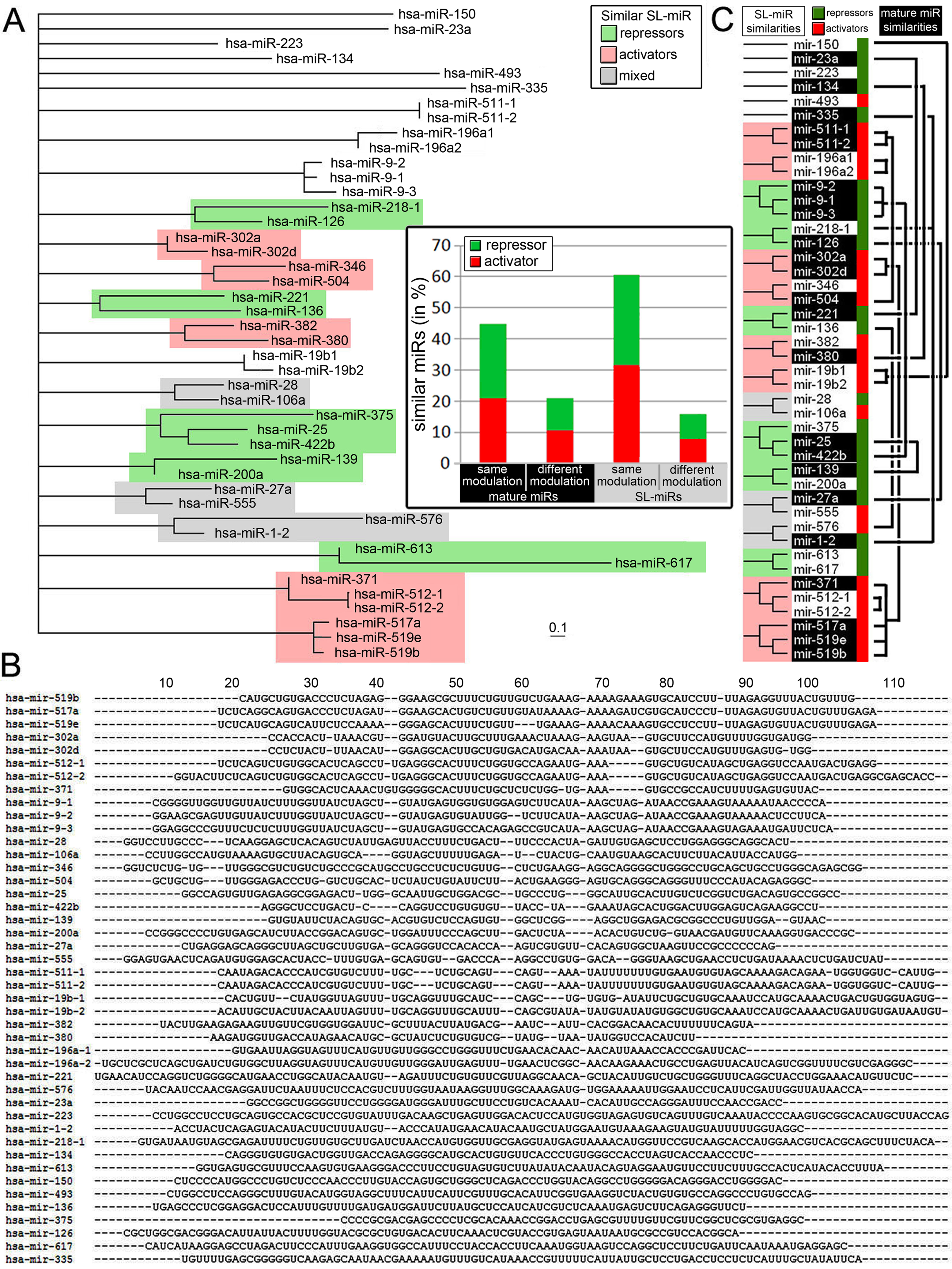

Supplement: Figure S3 — Alignment and phylogenetic quartet puzzling tree of all validated Wnt3a-modulating stem-loop miRs identified. (A) Phylogenetic tree of the alignment. Light green: Inhibitor Stem-Loop (SL)-miR similarity group. Light-red: activating SL-miR similarity group. Inset: Quantification of SL- and mature miRs that show the same effect on the canonical Wnt pathway. (B) Alignment of all validated Wnt-modulating SL-miR sequences using ClustalW. (C) Comparison and visualization of the similarity groups identified for stem-loop and mature miRs and their effect on the canonical Wnt pathway. (TIF) [file pone.0026257.s003.tif]

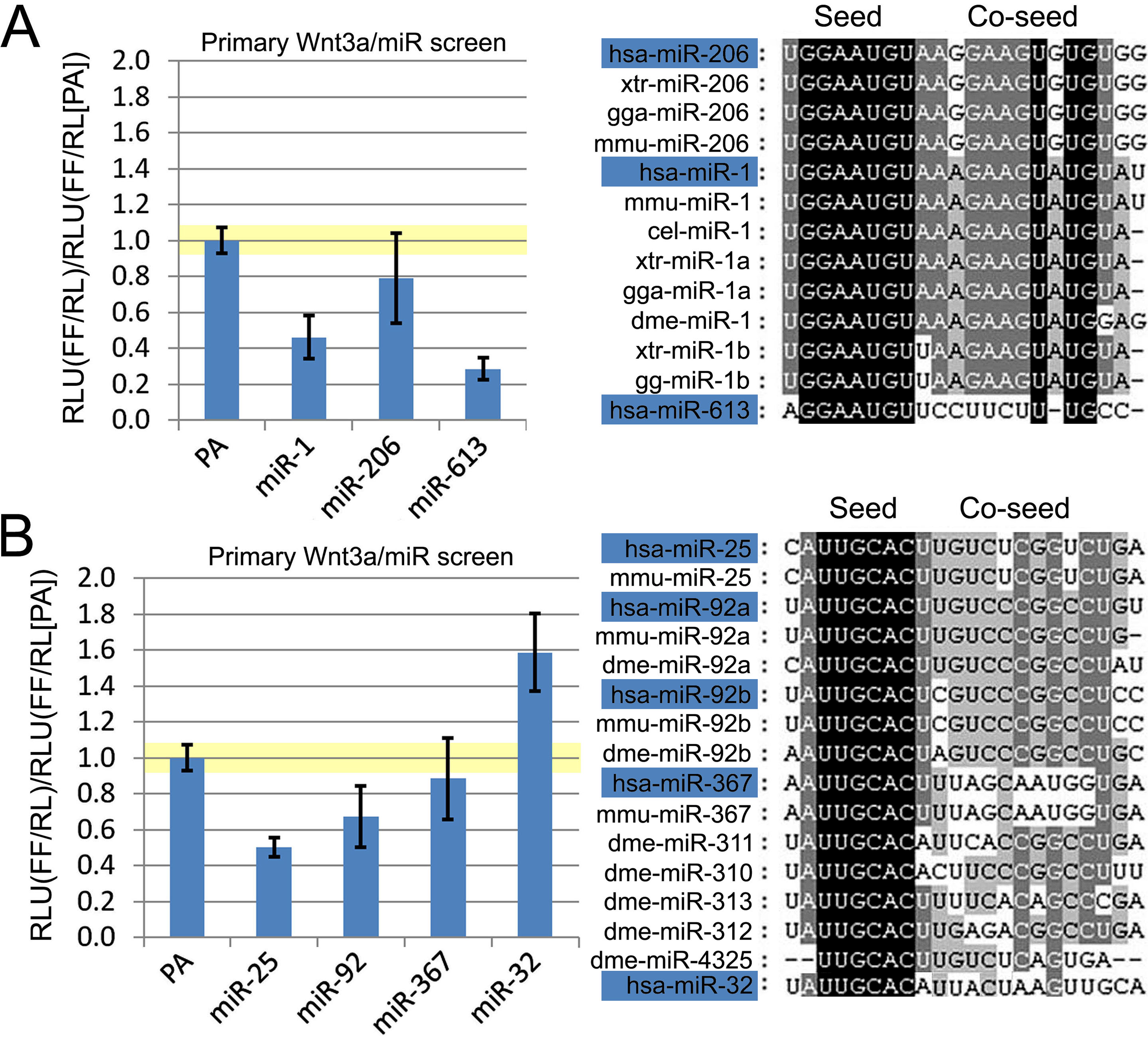

Supplement: Figure S4 — Screening results and alignment of studied miRs (miR-1/206 and miR-25/92 family) and miRs with a similar seed sequence. (A) STF19x/CMV-RL reporter values measured in the primary screen for miR-1/206 related miRs inheriting the GGAAUGU seed sequence and their alignment. (B) STF19x/CMV-RL reporter values measured in the primary screen for miR-25/92 related miRs inheriting the UUGCAC seed sequence and their alignment. Note that a few nucleotide substitutions could affect modulation of the Wnt-pathway as measured within the primary screen. (TIF) [file pone.0026257.s004.tif]

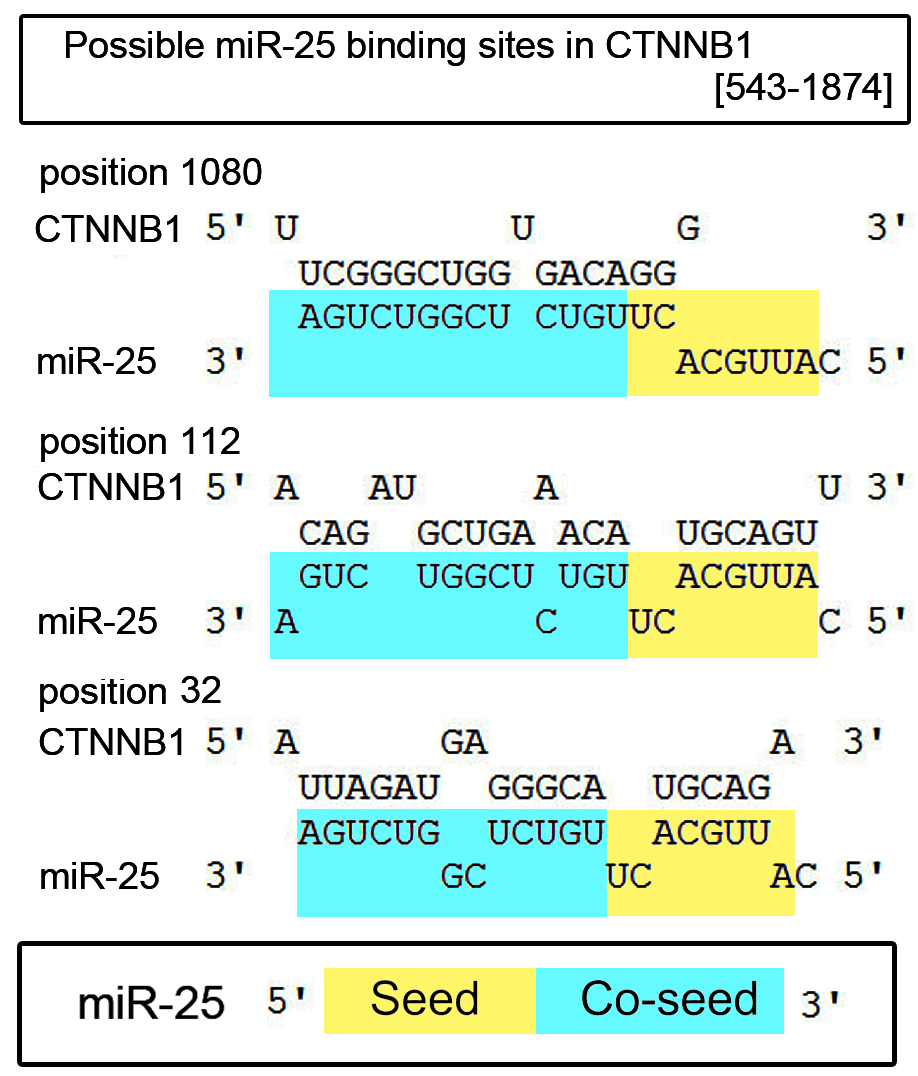

Supplement: Figure S5 — Possible miR-25 binding sites in β-catenin CDS (543–1874) predicted with RNAhybrid and without seed sequence constraints to include non-canonical seed identification. Mfe: minimum free energy is indicated. (TIF) [file pone.0026257.s005.tif]

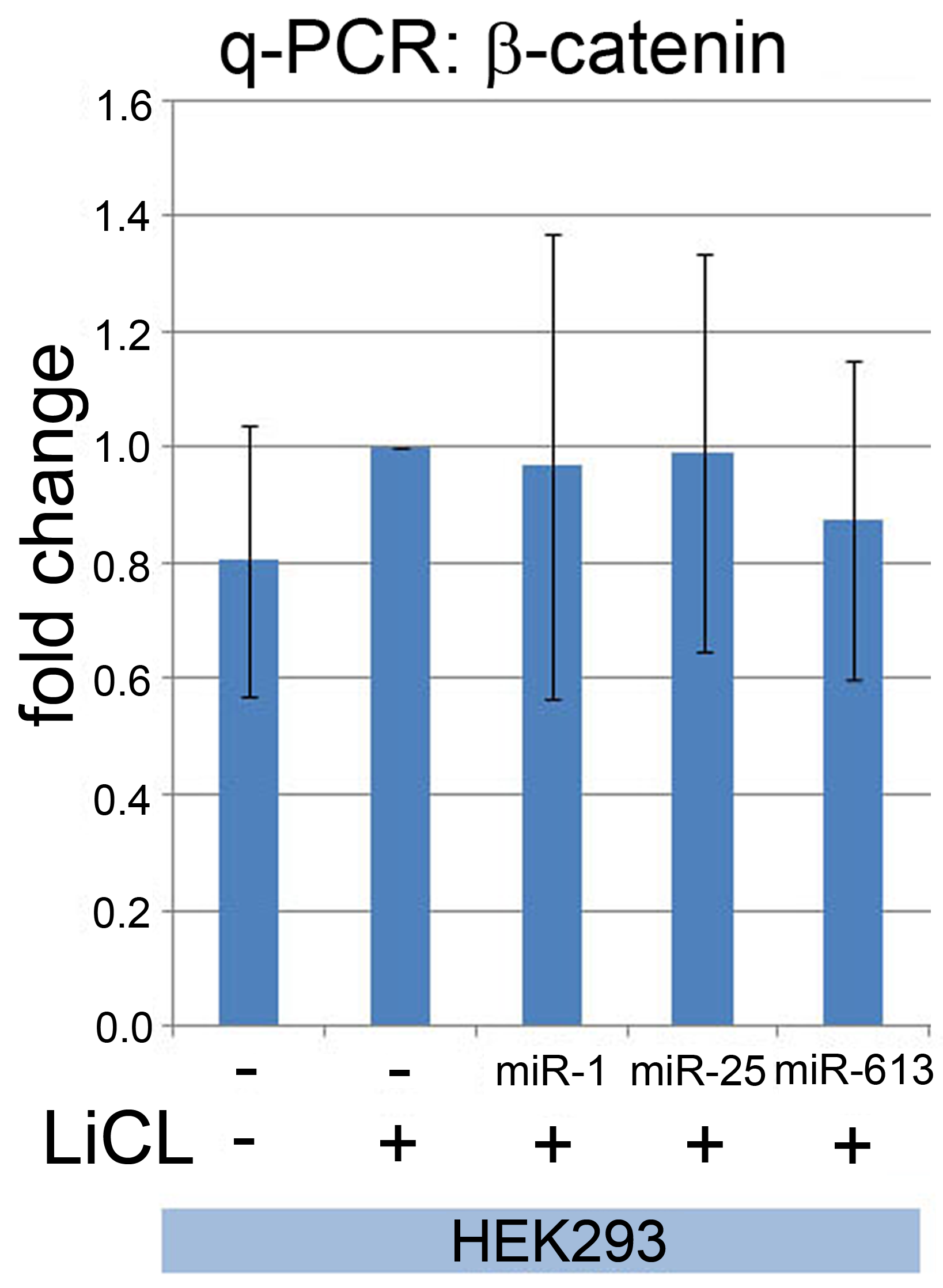

Supplement: Figure S7 — Q-PCR result for β-catenin mRNA levels in Hek293 cells transfected with 50 nM of indicated synthetic Pre-miRs or control siRNAs in the presence of 20 mM LiCl. Note: No significant changes of β-catenin mRNA levels could be measured for all miRs tested. (TIF) [file pone.0026257.s007.tif]

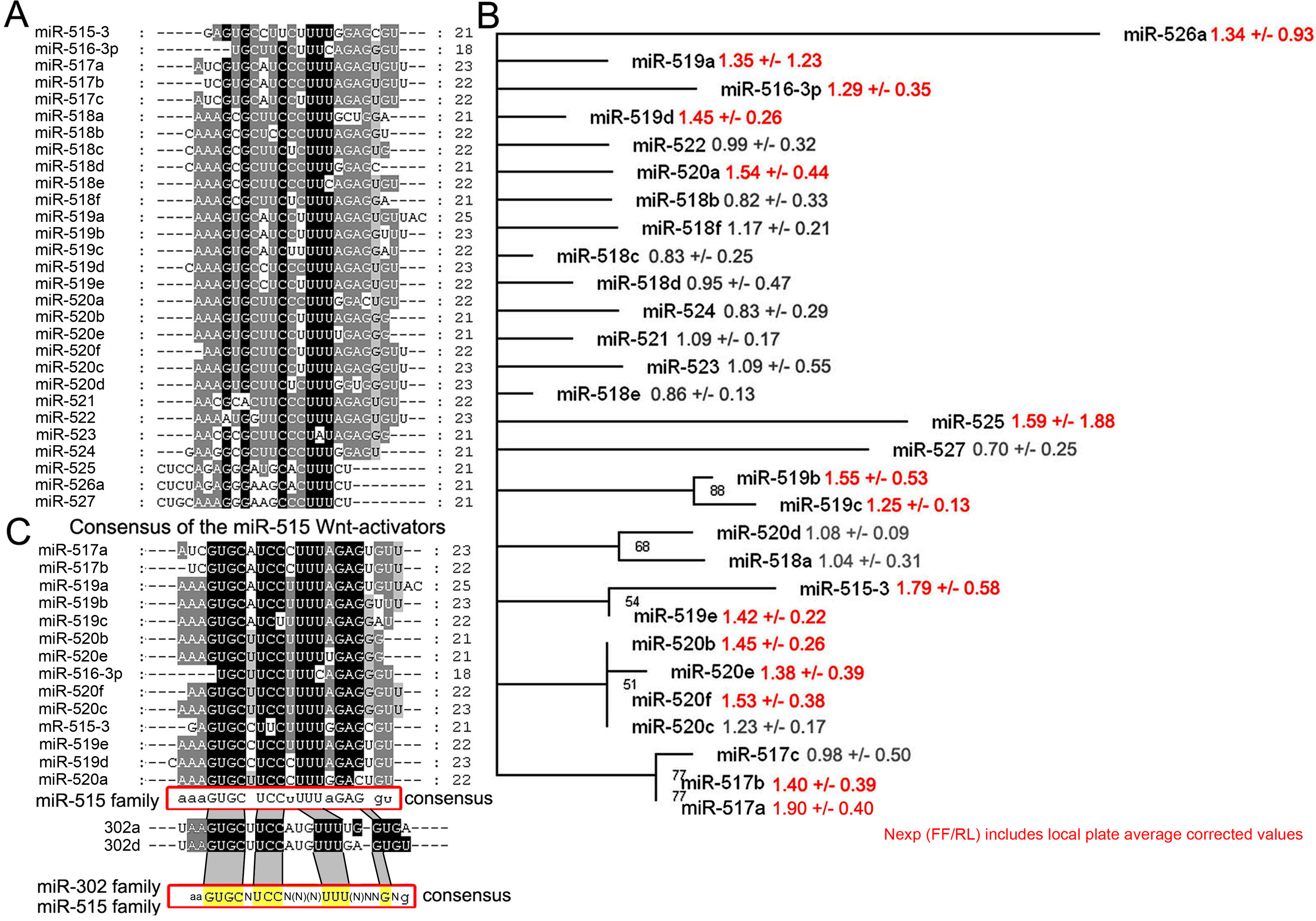

Supplement: Figure S8 — Extraction of a consensus sequence of identified miRs within the miR-515 and miR-302 family. Alignment to identify a functional consensus in the miR-515 family and its overlap with modulators the miR-302 family in regarding their ability to modulate the Wnt pathway. (A) Alignment of all tested miR-515 family members in the primary Wnt/miR-screen. (B) Tree of related miR-sequences with normalized Wnt/miR-screen values and standard deviations indicated (local plate average corrected). (C) Alignment of all isolated Wnt-synergizing miR-515 family members to identify a functional RNA consensus sequence that could be essential for the activation-potential on the canonical Wnt pathway. Identification of a common consensus sequence between the regulatory miR-515 and miR-203 family members (below). (TIF) [file pone.0026257.s008.tif]
