# Supplementary material for: A Systematic Screen for Micro-RNAs Regulating the Canonical Wnt Pathway
Source: PLoS One. 2011 Oct 17;6(10):e26257. doi: 10.1371/journal.pone.0026257 (PMC3197157; doi:10.1371/journal.pone.0026257)
Supplement: Figure/Table S6 — Table with all data mining references for the correlation studies of identified human Wnt-regulatory miRs and their oncogenicity. (T1.R) Oncogenicity of validated miR/Wnt-repressors. (T1.A) Oncogenicity of validated miR/Wnt-synergizer miRs. (T2.R) Expressional changes of validated miR/Wnt-repressors in cancer. (T2.A) Expression changes of validated miR/Wnt-synergizers in cancer. (DOCX) [file pone.0026257.s006.docx]

**Table 1-R** Oncogenicity of validated miRs – Wnt pathway repressors (n=20)

**miRNA Oncogenicity Carcinoma cell type Reference**

miR-218 anti-oncomiR Gastric Carcinoma [Tie et al., 2010](http://www.ncbi.nlm.nih.gov/pubmed/20300657)

anti-oncomiR Gastric Carcinoma [Gao et al., 2010](http://www.ncbi.nlm.nih.gov/pubmed/19890957)

miR-1 anti-oncomiR Rhabdomyosarcoma [Yan et al., 2009](http://www.ncbi.nlm.nih.gov/pubmed/19710019) [Rao et al., 2010](http://www.ncbi.nlm.nih.gov/pubmed/20466878)

anti-oncomiR Hepatocellular Carcinoma [Datta et al., 2008](http://www.ncbi.nlm.nih.gov/pubmed/?term=datta+microrna-1)

anti-oncomiR Colon Carcinoma (This study)

miR-613 not known

miR-126 anti-oncomiR Lung Carcinoma [Sun et al., 2009](http://www.ncbi.nlm.nih.gov/pubmed/20034472)

anti-oncomiR Gastric Carcinoma [Feng et al., 2010](http://www.ncbi.nlm.nih.gov/pubmed/20619534)

anti-oncomiR Breast Carcinoma [Tavazoie et al., 2008](http://www.ncbi.nlm.nih.gov/pubmed/18185580)

miR-25 [106b~25]oncomiR not known Hepatocellular Carcinoma [Li et al., 2009](http://www.ncbi.nlm.nih.gov/pubmed/19486339)

[106b~25]oncomiR not known Esophageal Carcinoma [Kan et al., 2009](http://www.ncbi.nlm.nih.gov/pubmed/19422085)

OncomiR Multiple Myeloma [Kumar et al., 2010](http://www.ncbi.nlm.nih.gov/pubmed/20935678)

OncomiR Prostate Carcinoma [Poliseno et al., 2010](http://www.ncbi.nlm.nih.gov/pubmed/20388916)

anti-oncomiR OncomiR Multiple Myeloma [Pichiorri et al., 2008](http://www.ncbi.nlm.nih.gov/pubmed/18728182)

anti-oncomiR not known Colon Carcinoma (This study)

miR-9* not known anti-oncomiR-sponge? Breast Carcinoma [Young et al., 2010](http://www.ncbi.nlm.nih.gov/pubmed/20173740)

miR-139 anti-oncomiR Hepatocellular Carcinoma [Wong et al., 2010](http://www.ncbi.nlm.nih.gov/pubmed/20951699)

miR-617 not known

miR-221 oncomiR Melanoma [Das et al., 2010](http://www.ncbi.nlm.nih.gov/pubmed/20547861)

oncomiR Breast Carcinoma [Leva et al., 2010](http://www.ncbi.nlm.nih.gov/pubmed/20388878)

oncomiR Leukemia [Frenquelli et al., 2010](http://www.ncbi.nlm.nih.gov/pubmed/20203269)

oncomiR Liver Carcinoma [Pineau et al., 2010](http://www.ncbi.nlm.nih.gov/pubmed/20018759)

oncomiR Hepatocellular Carcinoma [Fu et al., 2010](http://www.ncbi.nlm.nih.gov/pubmed/20146005)

oncomiR Pancreatic Carcinoma [Park et al., 2009](http://www.ncbi.nlm.nih.gov/pubmed/19730150)

oncomiR Prostate Carcinoma [Mercatelli et al., 2008](http://www.ncbi.nlm.nih.gov/pubmed/19107213)

oncomiR Lung Carcinoma [Garofalo et al., 2008](http://www.ncbi.nlm.nih.gov/pubmed/18246122)

oncomiR Breast Carcinoma [Sage et al., 2007](http://www.ncbi.nlm.nih.gov/pubmed/17627278)

oncomiR Prostate Carcinoma [Galardi et al., 2007](http://www.ncbi.nlm.nih.gov/pubmed/17569667)

miR-200a anti-oncomiR Breast Carcinoma [Volodymyr et al., 2009](http://www.ncbi.nlm.nih.gov/pubmed/19839049)

anti-oncomiR Nasopharyngeal Carcinoma [Xia et al., 2010](http://www.ncbi.nlm.nih.gov/pubmed/20826811) [Xia et al., 2010](http://www.ncbi.nlm.nih.gov/pubmed/19931509)

anti-oncomiR Brain Carcinoma [Saydam et al., 2009](http://www.ncbi.nlm.nih.gov/pubmed/19703993)

miR-223 anti-oncomiR Hepatocellular Carcinoma [Wong et al., 2008](http://www.ncbi.nlm.nih.gov/pubmed/18555017)

miR-23a [23a~27a]anti-oncomiR not known Embryonic Kidney [Chhabra et al., 2009](http://www.ncbi.nlm.nih.gov/pubmed/19513126)

oncomiR Gastric Carcinoma [Zhu et al., 2010](http://www.ncbi.nlm.nih.gov/pubmed/20698883)

anti-oncomiR Prostate Carcinoma [Gao et al., 2009](http://www.ncbi.nlm.nih.gov/pubmed/19219026)

[23a~27a]oncomiR not known Hepatocellular Carcinoma [Huang et al., 2008](http://www.ncbi.nlm.nih.gov/pubmed/18508316)

miR-150 oncomiR Gastric Carcinoma [Wu et al., 2010](http://www.ncbi.nlm.nih.gov/pubmed/20067763)

anti-oncomiR not known Leukemia [Lin et al., 2008](http://www.ncbi.nlm.nih.gov/pubmed/18667440)

miR-375 anti-oncomiR Gastric Carcinoma [Ding et al., 2010](http://www.ncbi.nlm.nih.gov/pubmed/20548334)

anti-oncomiR Liver Carcinoma [Liu et al., 2010](http://www.ncbi.nlm.nih.gov/pubmed/20226166)

miR-28 not known

miR-134 anti-oncomiR Lung Carcinoma [Guo et al., 2010](http://www.ncbi.nlm.nih.gov/pubmed/?term=guo+mir-134)

miR-27a oncomiR Breast Carcinoma [Yang et al., 2010](http://www.ncbi.nlm.nih.gov/pubmed/19921425)

oncomiR Gastric Carcinoma [Liu et al., 2009](http://www.ncbi.nlm.nih.gov/pubmed/18789835)

miR-136 not known

miR-422b not known

miR-335 anti-oncomiR Breast Carcinoma [Tavazoie et al., 2008](http://www.ncbi.nlm.nih.gov/pubmed/18185580)

---------------------------------------------------------------------------------------------------------------------------------------------------------------------------------------------------

**Anti-oncomiR = 9 (45.00%) of Wnt-repressors**

**OncomiR = 4 (20.00%) of Wnt-repressors**

**Unknown = 7 (35.00%) of Wnt-repressors**

**Unknown = Unknown/not known/not clear/as well as**

**Table 1-A** Oncogenicity of validated miRs – Wnt pathway activators (n=18)

**miRNA Oncogenicity Carcinoma cell type Reference**

miR-19b oncomiR Lymphoma [Mu et al., 2009](http://www.ncbi.nlm.nih.gov/pubmed/20008931)

oncomiR Lymphoma [Olive et al., 2009](http://www.ncbi.nlm.nih.gov/pubmed/20008935)

miR-512-3p not known

miR-519e anti-oncomiR Cervical carcinoma [Abdelmohsen et al., 2010](http://www.ncbi.nlm.nih.gov/pubmed/20305372)

miR-493-3p not known

miR-302a oncomiR Mammary Carcinoma [Borgdorff et al., 2009](http://www.ncbi.nlm.nih.gov/pubmed/20101223)

oncomiR miRPS [Lin et al., 2008](http://www.ncbi.nlm.nih.gov/pubmed/18755840)

miR-519b anti-oncomiR [Abdelmohsen et al., 2010](http://www.ncbi.nlm.nih.gov/pubmed/20305372)

anti-oncomiR Ovarian Carcinoma, Colon Carcinoma [Abdelmohsen et al., 2008](http://www.ncbi.nlm.nih.gov/pubmed/19088191)

miR-511 not known

miR-517a not known

miR-380-5p oncomiR Neuroblastoma, p53 [Swarbrick et al., 2010](http://www.ncbi.nlm.nih.gov/pubmed/?term=mir-380-5p)

miR-371 not known

miR-196a oncomiR Colorectal Carcinoma [Schimanski et al., 2009](http://www.ncbi.nlm.nih.gov/pubmed/19418581)

miR-346 oncomiR Follicular Thyroid Carcinoma [Weber et al., 2006](http://www.ncbi.nlm.nih.gov/pubmed/16822819)

miR-106a oncomiR Leukemia [Landais et al., 2007](http://www.ncbi.nlm.nih.gov/pubmed/17575136)

miR-555 not known

miR-576 not known

miR-382 not known

miR-504 oncomiR Carcinomas [Hu et al., 2010](http://www.ncbi.nlm.nih.gov/pubmed/20542001)

miR-302d oncomiR Mammary Carcinoma [Borgdorff et al., 2009](http://www.ncbi.nlm.nih.gov/pubmed/20101223)

---------------------------------------------------------------------------------------------------------------------------------------------------------------------------------------------------

**Anti-oncomiR = 2 (11.11%) of Wnt-activators**

**OncomiR = 8 (44.44%) of Wnt-activators**

**Unknown = 8 (44.44%) of Wnt-activators**

**Unknown = Unknown/not known/not clear/as well as**

**Table 2-R** Expression in Carcinoma – Wnt pathway repressors (n=20)

**miRNA Oncogenicity Expression Carcinoma cell type Reference .**

miR-218 anti-oncomiR Down Gastric carcinoma [Tie et al., 2010](http://www.ncbi.nlm.nih.gov/pubmed/?term=mir-218+robo1)

Down Gastric carcinoma [Gao et al., 2010](http://www.ncbi.nlm.nih.gov/pubmed/19890957)

Down Medulloblastoma [Lui et al., 2009](http://www.ncbi.nlm.nih.gov/pubmed/?term=mir-218+medulloblastoma)

Down Cervical papilloma carcinoma [Martinez et al., 2008](http://www.ncbi.nlm.nih.gov/pubmed/17998940)

Up Leukemia [Zanette et al., 2007](http://www.ncbi.nlm.nih.gov/pubmed/17934639)

Up Prostate carcinoma [Leite et al., 2009](http://www.ncbi.nlm.nih.gov/pubmed/19372056)

miR-1 anti-oncomiR Down Rhabdomyosarcoma [Yan et al., 2009](http://www.ncbi.nlm.nih.gov/pubmed/19710019) [Rao et al., 2010](http://www.ncbi.nlm.nih.gov/pubmed/20466878)

Down Colon carcinoma [Sarver et al., 2009](http://www.ncbi.nlm.nih.gov/pubmed/19922656)

miR-613 not known not known

miR-126 anti-oncomiR Down Breast, cervical carcinoma, leukemia [Sun et al., 2009](http://www.ncbi.nlm.nih.gov/pubmed/20034472)

Down Colorectal carcinoma [Li et al., 2009](http://www.ncbi.nlm.nih.gov/pubmed/20680522)

Down Gastric carcinoma [Feng et al., 2010](http://www.ncbi.nlm.nih.gov/pubmed/20619534)

Down Lung carcinoma [Nicoloso et al., 2009](http://www.ncbi.nlm.nih.gov/pubmed/19262572)

Bladder-, Prostate Carcinoma [Saito et al., 2009](http://www.ncbi.nlm.nih.gov/pubmed/19116145)

mirR-25 oncomiR Up Hepatocellular carcinoma [Li et al., 2009](http://www.ncbi.nlm.nih.gov/pubmed/19486339)

Up Prostate carcinoma [Poliseno et al., 2010](http://www.ncbi.nlm.nih.gov/pubmed/20388916)

Up Multiple Myeloma [Pichiorri et al., 2008](http://www.ncbi.nlm.nih.gov/pubmed/18728182)

miR-9* not known Down Colon carcinoma [Sarver et al., 2009](http://www.ncbi.nlm.nih.gov/pubmed/19922656)

miR-139 anti-oncomiR Down Liver carcinoma [Varnholt, 2008](http://www.ncbi.nlm.nih.gov/pubmed/18626426)

Down Esophageal carcinoma [Ogawa et al., 2009](http://www.ncbi.nlm.nih.gov/pubmed/19536617)

miR-617 not known Down Esophageal carcinoma [Yang et al., 2009](http://www.ncbi.nlm.nih.gov/pubmed/19737949)

miR-221 oncomiR Up Thyroid papillary carcinoma [Visone et al., 2007](http://www.ncbi.nlm.nih.gov/pubmed/17914108)

miR-200a anti-oncomiR Down Meningiomas [Saydam et al., 2009](http://www.ncbi.nlm.nih.gov/pubmed/19703993)

Up Pancreatic carcinomas [Li et al., 2010](http://www.ncbi.nlm.nih.gov/pubmed/20551052)

Down Gastric carcinomas [Shinozaki et al. 2010](http://www.ncbi.nlm.nih.gov/pubmed/20484038)

Down Bladder carcinomas [Wiklund et al., 2009](http://www.ncbi.nlm.nih.gov/pubmed/20473948)

Down Nasopharyngeal carcinoma [Xia et al., 2009](http://www.ncbi.nlm.nih.gov/pubmed/19931509)

miR-223 anti-oncomiR Down Hepatocellular carcinoma [Wong et al., 2008](http://www.ncbi.nlm.nih.gov/pubmed/18555017)

Down Lymphocytic leukemia [Stamatopoulos..., 2009](http://www.ncbi.nlm.nih.gov/pubmed/19144983)

miR-23a not known Down Prostate carcinoma [Porkka et al., 2008](http://www.ncbi.nlm.nih.gov/pubmed/17616669)

Up Down Diverse Carcinomas [Chabra et al., 2010](http://www.ncbi.nlm.nih.gov/pubmed/20815877)

miR-150 oncomiR Up Gastric carcinomas [Wu et al., 2010](http://www.ncbi.nlm.nih.gov/pubmed/20067763)

miR-375 anti-oncomiR Down Gastric carcinomas [Ding et al., 2010](http://www.ncbi.nlm.nih.gov/pubmed/20548334)

miR-28 not known Up Renal carcinomas [Gottardo et al., 2007](http://www.ncbi.nlm.nih.gov/pubmed/17826655sds)

miR-134 anti-oncomiR Down Colorectal carcinoma [Bandres et al., 2006](http://www.ncbi.nlm.nih.gov/pubmed/16854228)

miR-27a oncomiR Up Gastric carcinoma [Liu et al., 2009](http://www.ncbi.nlm.nih.gov/pubmed/18789835)

Up Ovarian carcinoma [Zhu et al., 2008](http://www.ncbi.nlm.nih.gov/pubmed/18619946)

miR-136 not known Up Lung carcinoma [Liu et al., 2010](http://www.ncbi.nlm.nih.gov/pubmed/20237410)

miR-422b not known Down Ovarian carcinoma [Lee et al., 2009](http://www.ncbi.nlm.nih.gov/pubmed/19798417)

miR-335 anti-oncomiR Down Breast carcinoma [Tavazoie et al., 2008](http://www.ncbi.nlm.nih.gov/pubmed/18185580)

---------------------------------------------------------------------------------------------------------------------------------------------------------------------------------------------------

**All Down = 12 (60.00%) of Wnt-repressors**

**All Up = 6 (30.00%) of Wnt-repressors**

**All Unknown = 2 (10.00%) of Wnt-repressors**

**Anti-oncomiR Down = 9 (45.00%) of Wnt-repressors**

**Anti-oncomiR up = 0 (0.00%) of Wnt-repressors**

**Anti-oncomiR Unknown = 0 (0.00%) of Wnt-repressors**

**oncomiR Down = 0 (0.00%) of Wnt-repressors**

**oncomiR up = 4 (20.00%) of Wnt-repressors**

**oncomiR Unknown = 0 (0.00%) of Wnt-repressors**

**Unknown Down = 3 (15.00%) of Wnt-repressors**

**Unknown up = 2 (10.00%) of Wnt-repressors**

**Unknown Unknown = 2 (10.00%) of Wnt-repressors**

**Unknown = Unknown/not known/not clear/as well as**

**Table 2-A** Expression in Carcinoma – Wnt pathway activators (n=18)

**miRNA Oncogenicity Expression Carcinoma type Reference** .

miR-19b oncomiR Up Breast, lung, colon carcinoma, B-cell lymphoma [Mu et al., 2009](http://www.ncbi.nlm.nih.gov/pubmed/20008931)

Up Medulloblastomas, Neuroblastoma, [Mu et al., 2009](http://www.ncbi.nlm.nih.gov/pubmed/20008931)

miR-512-3p not known not known atypical cervix dysplasia (up) [Pereira et al., 2010](http://www.ncbi.nlm.nih.gov/pubmed/20668671)

miR-519e anti-oncomiR Up Bladder carcinoma [Dyrskjot et al., 2009](http://www.ncbi.nlm.nih.gov/pubmed/19487295)

miR-493-3p not known Up Pancreatic carcinoma [Vandenboom.., 2008](http://www.ncbi.nlm.nih.gov/pubmed/19440450)

miR-302a oncomiR Up Colorectal carcinomas [Motoyama et al., 2008](http://www.ncbi.nlm.nih.gov/pubmed/19287964)

miR-519b anti-oncomiR Up Melanoma [Mueller et al., 2009](http://www.ncbi.nlm.nih.gov/pubmed/19212343)

miR-511 not known Down Adrenocortical carcinomas [Tömböl et al., 2009](http://www.ncbi.nlm.nih.gov/pubmed/19546168)

miR-517a not known Up Melanoma [Mueller et al., 2009](http://www.ncbi.nlm.nih.gov/pubmed/19212343)

miR-380-5p oncomiR Up Neuroblastoma [Swarbrick et al., 2010](http://www.ncbi.nlm.nih.gov/pubmed/?term=mir-380-5p)

miR-371 not known Up Thyroid Adenoma [Rippe et al., 2010](http://www.ncbi.nlm.nih.gov/pubmed/20209130)

Down Highly invasive Melanoma [Mueller et al., 2009](http://www.ncbi.nlm.nih.gov/pubmed/19212343)

Up Lung Carcinoma [Son et al., 2009](http://synapse.koreamed.org/Synapse/Data/PDFData/0003TRD/trd-67-413.pdf)

miR-196a oncomiR Up Colorectal carcinomas [Schimanski et al., 2009](http://www.ncbi.nlm.nih.gov/pubmed/19418581)

Up Esophageal carcinoma [Maru et al., 2009](http://www.ncbi.nlm.nih.gov/pubmed/19342367)

Up Pancreatic carcinomas [Bloomston et al., 2007](http://www.ncbi.nlm.nih.gov/pubmed/17473300)

miR-346 oncomiR Up Follicular thyroid carcinoma [Weber et al., 2006](http://www.ncbi.nlm.nih.gov/pubmed/16822819)

miR-106a oncomiR Up Colorectal carcinoma [Link et al., 2010](http://www.ncbi.nlm.nih.gov/pubmed/20551304)

Up Gastric carcinomas [Guo et al., 2005](http://www.ncbi.nlm.nih.gov/pubmed/19175831) Up Gastric Carcinoma [Xiao et al., 2009](http://www.ncbi.nlm.nih.gov/pubmed/18996365)

miR-576 not known not known Highly invasive Gastric Carcinoma [Tie et al., 2010](http://www.ncbi.nlm.nih.gov/pubmed/20300657)

miR-555 not known not known Colon Carcinoma [Sarver et al., 2009](http://www.ncbi.nlm.nih.gov/pubmed/19922656)

miR-382 not known Up Acute myeloid leukemia [Jongen-...2008](http://www.ncbi.nlm.nih.gov/pubmed/18337557)

miR-504 oncomiR not known

miR-302d oncomiR Up Germinal cell carcinomas [Palmer et al., 2010](http://www.ncbi.nlm.nih.gov/pubmed/20332240)

---------------------------------------------------------------------------------------------------------------------------------------------------------------------------------------------------

**All Down = 1 (5.56%) of Wnt-activators**

**All Up = 13 (72.22%) of Wnt-activators**

**All Unknown = 4 (22.22%) of Wnt-activators**

**Anti-oncomiR Down = 0 (0.00%) of Wnt-activators**

**Anti-oncomiR up = 2 (11.11%) of Wnt-activators**

**Anti-oncomiR Unknown = 0 (0.00%) of Wnt-activators**

**oncomiR Down = 0 (0.00%) of Wnt-activators**

**oncomiR up = 7 (38.89%) of Wnt-activators**

**oncomiR Unknown = 1 (5.56%) of Wnt-activators**

**Unknown Down = 1 (5.56%) of Wnt-activators**

**Unknown up = 4 (22.22%) of Wnt-activators**

**Unknown Unknown = 3 (16.67%) of Wnt-activators**

**Unknown = Unknown/not known/not clear/as well as**
